# Plasmodesmal connectivity in C_4_ *Gynandropsis gynandra* is induced by light and dependent on photosynthesis

**DOI:** 10.1111/nph.19343

**Published:** 2023-10-26

**Authors:** Tina B. Schreier, Karin H. Müller, Simona Eicke, Christine Faulkner, Samuel C. Zeeman, Julian M. Hibberd

**Affiliations:** ^1^ Department of Plant Sciences University of Cambridge Downing Street Cambridge CB1 3EA UK; ^2^ Cambridge Advanced Imaging Centre (CAIC) University of Cambridge Downing Street Cambridge CB2 3DY UK; ^3^ Institute of Molecular Plant Biology ETH Zurich Zurich CH‐8092 Switzerland; ^4^ Cell and Developmental Biology John Innes Centre Norwich Research Park Norwich NR4 7UH UK; ^5^ Present address: Department of Biology University of Oxford South Parks Road Oxford OX1 3RB UK

**Keywords:** bundle sheath, C_4_ photosynthesis, light, mesophyll, photomorphogenesis, photosynthesis, plasmodesmata

## Abstract

- In leaves of C_4_ plants, the reactions of photosynthesis become restricted between two compartments. Typically, this allows accumulation of C_4_ acids in mesophyll (M) cells and subsequent decarboxylation in the bundle sheath (BS). In C_4_ grasses, proliferation of plasmodesmata between these cell types is thought to increase cell‐to‐cell connectivity to allow efficient metabolite movement. However, it is not known whether C_4_ dicotyledons also show this enhanced plasmodesmal connectivity and so whether this is a general requirement for C_4_ photosynthesis is not clear. How M and BS cells in C_4_ leaves become highly connected is also not known.
- We investigated these questions using 3D‐ and 2D‐electron microscopy on the C_4_ dicotyledon *Gynandropsis gynandra* as well as phylogenetically close C_3_ relatives.
- The M–BS interface of C_4_ *G. gynandra* showed higher plasmodesmal frequency compared with closely related C_3_ species. Formation of these plasmodesmata was induced by light. Pharmacological agents that perturbed photosynthesis reduced the number of plasmodesmata, but this inhibitory effect could be reversed by the provision of exogenous sucrose.
- We conclude that enhanced formation of plasmodesmata between M and BS cells is wired to the induction of photosynthesis in C_4_ *G. gynandra*.

## Introduction

C_4_ photosynthesis represents a carbon‐concentrating mechanism that has repeatedly evolved from the ancestral C_3_‐type of photosynthesis (Sage *et al*., 2011). In leaves of C_4_ plants, HCO_3_^−^ is initially fixed by phopsho*enol*pyruvate carboxylase (PEPC) in mesophyll (M) cells into a 4‐carbon acid oxaloacetate that is then converted to aspartate or malate. These C_4_ acids then move to bundle sheath (BS) cells for decarboxylation to produce pyruvate and CO_2_ (or PEP and CO_2_ in the case of the PEP carboxykinase (PCK) subtype of C_4_ photosynthesis). Pyruvate is transferred back to the M cells where it is reduced to phospho*enol*pyruvate that can accept another HCO_3_^−^ molecule. This spatial separation of carboxylation and decarboxylation between M and BS cells builds a high concentration of CO_2_ in BS cells and in so doing limits the oxygenation side‐reaction of RuBisCO (Hatch, 1987). This greatly increases photosynthesis efficiency, particularly in hot and dry environments.

Efficient exchange of metabolites between M and BS cells is therefore crucial to the C_4_ pathway and, as a consequence, compared with the ancestral C_3_ condition C_4_ leaves are typically reconfigured in both biochemistry and structure. Most C_4_ plants have Kranz anatomy characterized by closely spaced veins and a wreath‐like, concentric arrangement of enlarged BS cells surrounding M cells that maximizes M–BS contact sites (Sedelnikova *et al*., 2018). Kranz anatomy is associated with increased cell‐to‐cell connectivity between the M and BS cells to allow the efficient exchange of metabolites. Metabolite exchange between the two cell types is proposed to occur via passive diffusion through plasmodesmata down a steep concentration gradient of C_4_ metabolites (Hatch, 1987).

Plasmodesmata are regulated channels between adjacent plant cells and are diverse in structure: from simple (with single openings in adjacent cells) to complex (highly branched with central cavities), or even asymmetric in their organization (Ross‐Elliott *et al*., 2017; Faulkner, 2018). Plasmodesmata contain several structural components including a narrow tube of endoplasmic reticulum called the desmotubule, the cytoplasmic sleeve and the plasma membrane (Faulkner, 2018). They can either be formed *de novo* during cell division by trapping endoplasmic reticulum strands between enlarging Golgi‐derived vesicles in new cell walls (primary plasmodesmata) or formed in pre‐existing cell walls (secondary plasmodesmata; Hepler, 1982; Ehlers & Kollmann, 2001; Faulkner *et al*., 2008). Plasmodesmata are considered essential for cell‐to‐cell transport of metabolites in many C_4_ grasses because suberized BS cell walls likely reduce CO_2_ leakage by blocking apoplastic metabolite transfer (Hatch & Osmond, 1976). Furthermore, C_4_ grasses possess increased numbers of plasmodesmata between M and BS cells (Evert *et al*., 1977; Botha, 1992; Botha *et al*., 1993; Danila *et al*., 2016). As plasmodesmata occur in clusters (pit fields), increased cell‐to‐cell connectivity in C_4_ leaves can be a result of increased pit field area or increased numbers of plasmodesmata per pit field area. Danila *et al*. (2016) observed up to ninefold increase in plasmodesmal frequency at the M–BS interface in C_4_ maize and *Setaria viridis* compared with the C_3_ species rice and wheat. This increase in the C_4_ grasses was due to a twofold increase in plasmodesmata numbers per pit field and a fivefold increase in pit field area. In other C_4_ grasses, substantial variation in absolute plasmodesmata frequency was evident, but in all cases, they possessed greater plasmodesmata frequency than C_3_ species (Danila *et al*., 2018).

To our knowledge, the distribution of plasmodesmata at the M–BS cell interface of C_3_ and C_4_ species has not been studied outside the grasses. Furthermore, the cues that underpin increased plasmodesmata formation are not known. Given the known variation in how increased cell‐to‐cell connectivity is achieved in C_4_ grasses and the fact that they evolved C_4_ photosynthesis independently from C_4_ dicotyledonous lineages, we assessed plasmodesmata distribution in leaves of C_3_ *Tarenaya hassleriana* and C_4_ *Gynandropsis gynandra* that both belong to the *Cleomaceae* (Brown *et al*., 2005; Marshall *et al*., 2007), which is sister to the *Brassicaceae*. *G. gynandra* has been adopted as a C_4_ model (Brown *et al*., 2005; Marshall *et al*., 2007; Bräutigam *et al*., 2011; Koteyeva *et al*., 2011). We discovered that plasmodesmal frequency is up to 13‐fold higher at the M–BS cell interface in mature leaves of C_4_ *G. gynandra* compared with that in C_3_ species. Moreover, these increased numbers of plasmodesmata are rapidly established during de‐etiolation. Pharmacological studies using multiple chloroplast inhibitors demonstrated that light, functional chloroplasts and photosynthesis are required to initiate plasmodesmata formation at M–BS cell interface of *G. gynandra*. Provision of exogenous sucrose can rescue defects in chloroplasts and photosynthesis. We conclude that increased plasmodesmatal connection is likely an unifying feature of all two‐celled C_4_ plants, and that during the evolution of the C_4_ pathway the increased formation of secondary plasmodesmata is stimulated by the induction of photosynthesis itself.

## Materials and Methods

### Plant material and growth conditions

*Gynandropsis gynandra* (L.) and *Tarenaya hassleriana* (Chodat) seeds were germinated on wet filter papers in Petri dishes. For *G. gynandra*, germination was initiated by exposing seeds to 30°C for 24 h. For *T. hassleriana*, germination was stimulated by an alternating temperature regime of 12 h at 32°C then 12 h at 20°C for 5 consecutive days. After germination, *G. gynandra* and *T. hassleriana* seedlings were planted in individual pots in 10 : 1 ratio of M3 compost (Levington Advance, Pot and Bedding, High Nutrient) to fine vermiculite. *Arabidopsis thaliana* (L.) (Col‐0) was sown onto potting compost (Levington Advance Solutions, ICL Group Ltd, Doncaster, UK) with 0.17 g L^−1^ insecticide (thiacloprid, Exemptor, ICL Group Ltd) and stratified for 48 h at 4°C. Around 2 wk after germination, individual seedlings were transplanted to individual pots.

To sample mature leaves of 4‐wk‐old *G. gynandra* and *T. hassleriana* plants and 3‐wk‐old Arabidopsis plants, they were grown in a climate‐controlled growth chamber with 16 h : 8 h, light : dark. *G. gynandra* and *T. hassleriana* were grown at 350 μmol photons m^−2^ s^−1^ at 25°C with a 60% (v/v) relative humidity and ambient CO_2_. *Arabidopsis thaliana* plants were grown under identical conditions except light intensity was 150 μmol photons m^−2^ s^−1^. All plants were watered by an automated system whereby the bottom of the trays was flooded to a depth of *c*. 4 cm every 48 h for 10 min, after which the irrigation water was drained.

For de‐etiolation experiments, *G. gynandra* seeds were germinated with the addition of 0.15% (v/v) plant preservative mixture (CAS: 26172‐55‐4; Apollo Scientific, Bredbury, UK) to the wet filter paper. Germinated seedlings were transferred to square plates containing ½‐strength Murashige & Skoog medium (½MS) salts with B5 vitamins (Duchefa Biochemie B.V., Haarlem, Netherlands) and 0.8% (w/v) agar (Melford, Ipswich, UK) in the dark. Plates were grown in the plant growth cabinet (MLR‐352 PE; Panasonic) at 20°C with continuous light intensity of 100 μmol m^−2^ s^−1^. Plates were covered with aluminum foil for 3 consecutive days to ensure no light was able to penetrate. Foil was removed on Day 3 and to allow de‐etiolation and plants were grown for an additional 24–48 h in the light. For sucrose supplementation, 10 g l^−1^ sucrose was added to the ½MS media. For inhibitor treatments, 500 μM lincomycin (Sigma Aldrich), 50 μM norflurazon (Sigma‐Aldrich) and 20 μM 3‐(3,4‐dichlorophenyl)‐1,1‐dimethylurea (DCMU; Sigma‐Aldrich) were added to the ½MS before the media was poured in the individual Petri dishes. As norflurazon and lincomycin were dissolved in ethanol, the control and DCMU treatments included an equivalent amount of ethanol in the media.

### Sample preparation for electron microscopy

To assess mature leaves, samples from four to six individual plants for each species were taken. For the de‐etiolation experiment of *G. gynandra*, samples from five to eight individual seedlings at each time point (0, 24 and 48 h) were harvested for electron microscopy. Leaf segments (*c*. 2 mm^2^) were excised with a razor blade and immediately fixed in 2% (v/v) glutaraldehyde and 2% (w/v) formaldehyde in 0.05–0.1 M sodium cacodylate (NaCac) buffer (pH 7.4) containing 2 mM calcium chloride. Samples were vacuum infiltrated overnight, washed five times in 0.05–0.1 M NaCac buffer and postfixed in 1% (v/v) aqueous osmium tetroxide and 1.5% (w/v) potassium ferricyanide in 0.05 M NaCac buffer for 3 d at 4°C. After osmication, samples were washed five times in deionized water and postfixed in 0.1% (w/v) thiocarbohydrazide for 20 min at room temperature in the dark. Samples were then washed five times in deionized water and osmicated for a second time for 1 h in 2% (v/v) aqueous osmium tetroxide at room temperature. Samples were washed five times in deionized water and subsequently stained in 2% (w/v) uranyl acetate in 0.05 M maleate buffer (pH 5.5) for 3 d at 4°C and washed five times afterward in deionized water. Samples were then dehydrated in an ethanol series, transferred to acetone and then to acetonitrile. Leaf samples were embedded in Quetol 651 resin mix (TAAB Laboratories Equipment Ltd, Aldermaston, UK) and cured at 60°C for 2 d.

### Transmission electron microscopy and scanning electron microscopy

For transmission electron microscopy (TEM), ultra‐thin sections were cut with a diamond knife using a Leica Ultracut microtome and collected on copper grids and examined in a FEI Tecnai G2 transmission electron microscope (200 keV, 20 μm objective aperture). Images were obtained with an AMT CCD camera.

For 2D SEM mapping, ultra‐thin sections were placed on Melinex (TAAB Laboratories Equipment Ltd) plastic coverslips mounted on aluminum SEM stubs using conductive carbon tabs (TAAB Laboratories Equipment Ltd), sputter‐coated with a thin layer of carbon (*c*. 30 nm) to avoid charging and imaged in a Verios 460 scanning electron microscope at 4 keV accelerating voltage and 0.2 nA probe current using the concentric backscatter detector in field‐free (low magnification) or immersion (high magnification) mode (working distance 3.5–4 mm, dwell time 3 μs, 1536 × 1024 pixel resolution). For plasmodesmata frequency quantification, SEM stitched maps were acquired at 10 000× magnification using the FEI MAPS automated acquisition software. Greyscale contrast of the images was inverted to allow easier visualization.

Serial block‐face scanning electron microscopy (SBF‐SEM) was performed on Quetol 651 resin‐embedded mature leaf samples of *G. gynandra*, *T. hassleriana* and *A. thaliana* as described above. Overviews of leaf cross‐sections and the zoomed stacks of the M–BS cell interface (*c*. 300–400 images) were acquired through sequentially sectioning the block faces at 50 nm increments and imaging the resulting block face by SEM. Images were acquired with a scanning electron microscope TFS Quanta 250 3VIEW (FEI, Hillsboro, OR, USA) at 1.8–2 keV with an integrated 3VIEW stage and a backscattered electron detector (Gatan Inc., Pleasanton, CA, USA). Images were aligned and smoothed using the plugins MultiStackReg and 3D median filter on imagej. A subset of 80 sections was used for 3D reconstruction of the M–BS cell interface in each of the three datasets. Structural features (cell wall and individual plasmodesmata) were then marked manually with Amira (Thermo Fisher, Waltham, MA, USA).

### Plasmodesmata quantification

Plasmodesmal frequency from 2D and 3D EM images was determined using published methods (Botha, 1992; Koteyeva *et al*., 2014). Briefly, plasmodesmal frequency was determined as the number of plasmodesmata observed per μm of length of shared cell interface between two cell types (M–BS, M–M and BS–BS). Plasmodesmata numbers and cell lengths were determined using imagej software. Plasmodesmata were defined as dark channels in the EM images. Depending on plasmodesmata orientation, the entire channel was sometimes not visible on 2D EM images, and so only channels that spanned more than half of the cell wall width were counted.

### Chlorophyll fluorescence measurement

Chlorophyll fluorescence measurements were carried out using a CF imager (Technologica Ltd, Colchester, UK), and image processing software was provided by the manufacturer. Seedlings were placed in the dark for 20 min, and a minimum weak measuring light beam (<1 μmol m^−2^ s^−1^) was applied to evaluate dark‐adapted minimum fluorescence (*F*_o_), and a subsequent saturating pulse of 6000 μmol m^−2^ s^−1^ was applied to evaluate dark‐adapted maximum fluorescence (*F*_m_), and then, variable fluorescence *F*_v_ was calculated according to the following formula: *F*_v_ *= F*_m_ *–F*_o_. All chlorophyll fluorescence images of inhibitor‐treated seedlings within each experiment were acquired at the same time in a single image, measuring three to eight seedlings per treatment.

### Statistical analysis

In violin plots, the middle line represents the median, the box and whiskers represent the 25–75 percentile and minimum–maximum distributions of the data, respectively. Letters show the statistical ranking using a one‐way ANOVA and *post hoc* Tukey test (different letters indicate differences at *P* < 0.05). Values indicated by the same letter are not statistically different. Data were analyzed using rstudio 2022.07.2 + 576.

## Results

### Plasmodesmata frequency is higher in C_4_ *G. gynandra* leaves compared with C_3_ *A. thaliana* and *T. hassleriana*

**Fig. 1:**
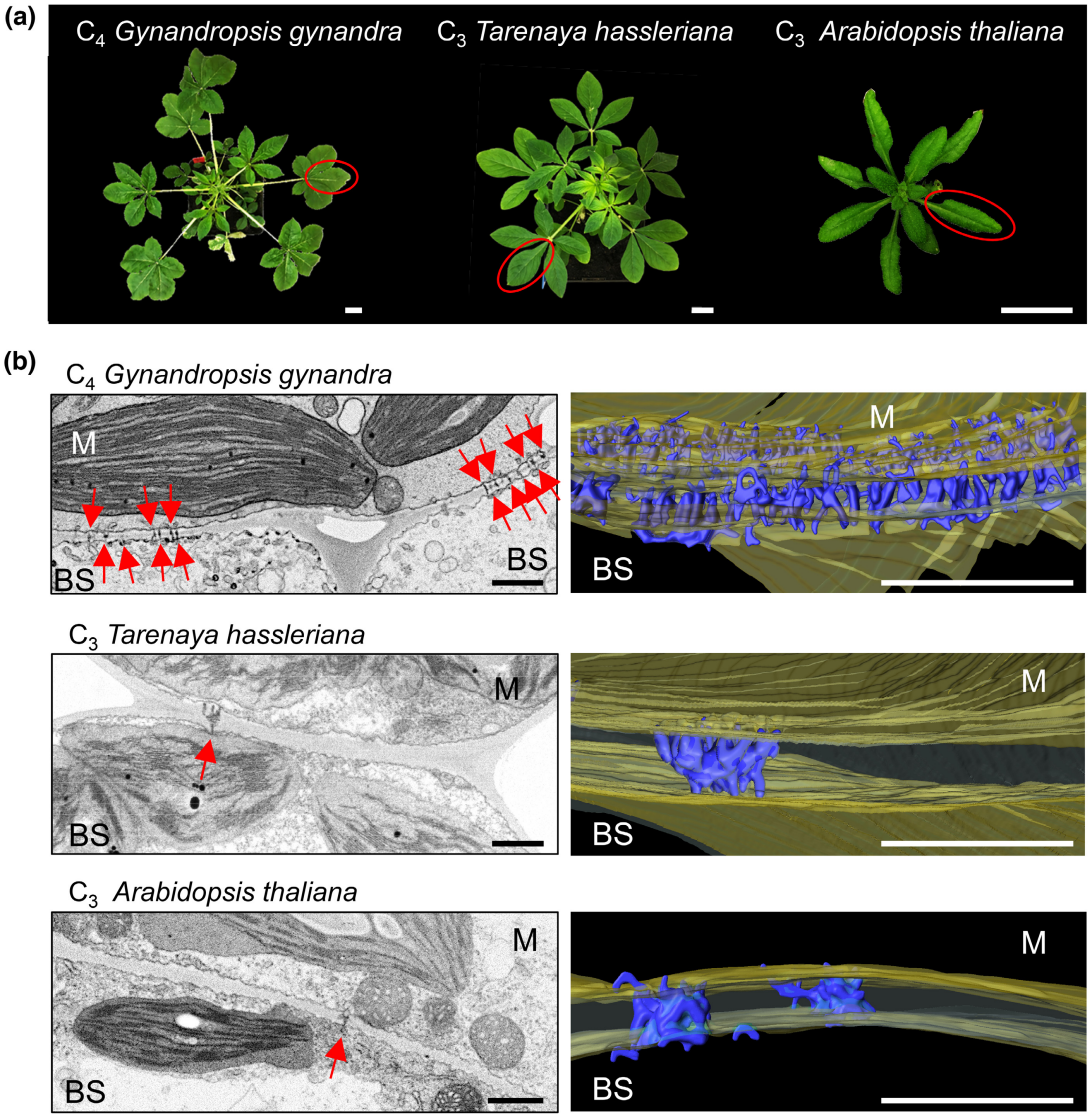
The mesophyll (M)–bundle sheath (BS) cell interface of C_4_ *Gynandropsis gynandra* has more plasmodesmata than closely related C_3_ species. (a) Photographs of 4‐wk‐old C_4_ *G. gynandra* (left) and C_3_ *Tarenaya hassleriana* (middle) and 3‐wk‐old C_3_ *Arabidopsis thaliana* plants (right). Mature leaves harvested for plasmodesmata quantification are circled in red. Bars, 2 cm. (b) Left panels: representative transmission electron micrographs of M and BS cell interface in all three species. Red arrows indicate individual plasmodesma. Bars, 1 μm. Right panels: 3D reconstructions of cell interfaces from serial block‐face scanning electron microscopy (SBF‐SEM; see Supporting Information Videos [Link], [Link]). Cell walls are colored in yellow and individual plasmodesmata in blue. Bars, 1 μm.

**Fig. 2:**
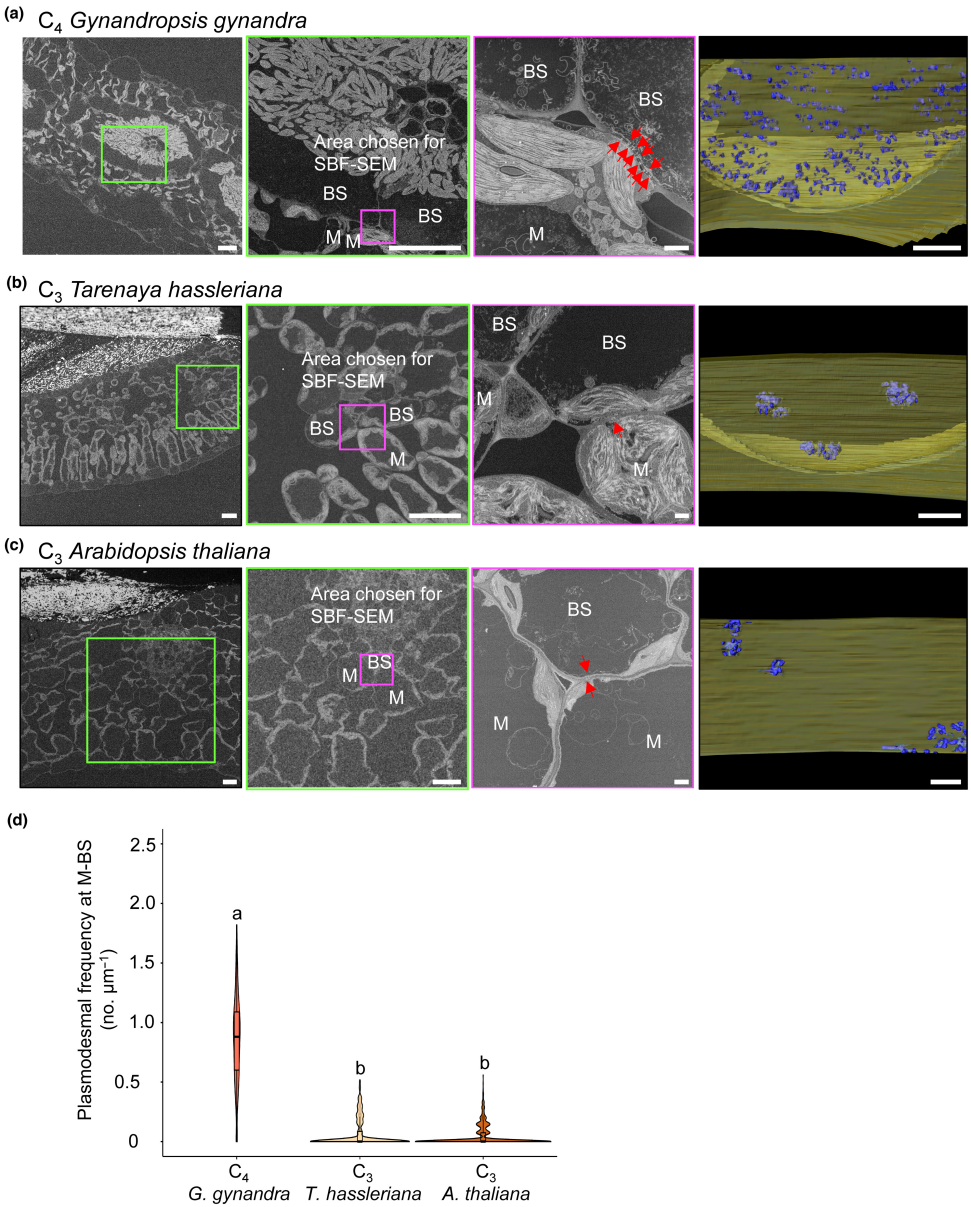
3D Serial block‐face scanning electron microscopy (SBF‐SEM) analysis of plasmodesmata number at the mesophyll (M)–bundle sheath (BS) cell interface. Left panels: Representative scanning electron micrographs of leaf cross‐sections from (a) C_4_ *Gynandropsis gynandra*, (b) C_3_ *Tarenaya hassleriana* and (c) C_3_ *Arabidopsis thaliana*. Bars, 20 μm. Mid left panels: zoomed in image of the region marked by a green box showing area of M and BS cell interface used for SBF‐SEM analysis (magenta). Bars, 20 μm. Mid right panels: single frame of compiled SBF‐SEM data into Supporting Information Videos [Link], [Link] of (a) C_4_ *G. gynandra*, (b) C_3_ *T. hassleriana* and (c) C_3_ *A. thaliana*. Red arrows indicate individual plasmodesma. Bars, 1 μm. Right panels: 3D reconstructions of M–BS cell interface from SBF‐SEM (Supporting Information Videos [Link], [Link]) of (a) C_4_ *G. gynandra*, (b) C_3_ *T. hassleriana* and (c) C_3_ *A. thaliana*. 3D reconstruction is depicted from each BS cell looking through to the M cell, and the cell wall is colored in yellow and individual plasmodesmata in blue. Bars, 1 μm. (d) Violin plot of plasmodesmal frequencies measured at M–BS cell interfaces in the three plant species using 3D SBF‐SEM data. As some sections contained more than one cell interface, plasmodesmata frequencies were quantified from a total of 467 individual M–BS cell interfaces for *G. gynandra*, 367 for *T. hassleriana* and 886 for *A. thaliana*. Box and whiskers represent the 25 to 75 percentile and minimum–maximum distributions of the data. Letters show statistical ranking using a *post hoc* Tukey test (with different letters indicating statistically significant differences at *P* < 0.05). Values indicated by the same letter are not statistically different.

We first explored whether the increased plasmodesmal connectivity between M and BS cells found in C_4_ grasses was also present in the C_4_ dicotyledon *Gynandropsis gynandra*. Transmission electron microscopy was used to examine the M–BS cell interface in mature leaves of *G. gynandra* and the closely related C_3_ species *Tarenaya hassleriana* (also a member of the *Cleomaceae*) and C_3_ *Arabidopsis thaliana* (Fig. 1a). Plasmodesmata were more abundant between M and BS cells in C_4_ *G. gynandra* compared with both C_3_ species (Fig. 1a,b). We used SBF‐SEM to visualize these plasmodesmata in 3D. Thin sections from fully expanded true leaves of *G. gynandra*, *T. hassleriana* and *A. thaliana* were imaged, and an area of the M–BS cell interface was identified for serial block‐face sectioning (Fig. 2a–c). For each species, a subset of sections (*n* = 80) was then used for 3D reconstructions of the cell interface by hand‐segmentation of the cell wall (in yellow) and individual plasmodesmata structures (in blue) (Fig. 1b; Supporting Information Videos [Link], [Link]). Consistent with the TEM images, 3D reconstructions showed increased plasmodesmal connectivity between M and BS cells in the C_4_ species compared with the C_3_ species (Fig. 1b; Videos [Link], [Link]). Greater physical connectivity was specific to this interface, and no obvious differences in plasmodesmata number were detected at the M–M or BS–BS cell interfaces between these species (Fig. S1).

To quantify plasmodesmata numbers between M and BS cells, we used these SBF‐SEM data, an approach that has previously been used to quantify plasmodesmata in other systems (Ross‐Elliott *et al*., 2017; Paterlini & Belevich, 2022). The full SBF‐SEM stacks containing between 281 and 438 serial transverse sections per M–BS cell interface were used to quantify plasmodesmata frequency by determining the number of plasmodesmata per unit M–BS cell interface imaged (Fig. 2d) and then compiled into videos (Videos [Link], [Link]). In C_4_ *G. gynandra*, plasmodesmata were visible in almost every M–BS cell interface assessed such that only 20 of the 467 imaged contained no plasmodesmata (Fig. 2d). By contrast, in the two C_3_ species plasmodesmata were not detected in the majority of interfaces (263/367 for *T. hassleriana*, 628/886 for *A. thaliana*). Plasmodesmata can appear in clusters (pit fields) rather than being equally distributed, and thus, a wide range of plasmodesmal frequencies per section were observed between M and BS cells in all three species. However, there were more sections with higher frequencies observed at the M–BS interface of C_4_ *G. gynandra*, and this resulted in a 13‐fold increase in the mean frequency compared with C_3_ *T. hassleriana* and C_3_ *A. thaliana* (Fig. 2d). Plasmodesmal frequencies between M and BS cells of the C_3_ species *T. hassleriana* and *A. thaliana* were not significantly different to each other and were low compared with C_4_ *G. gynandra*. To assess plasmodesmata distribution in more detail, we utilized the 3D reconstructions generated from the SBF‐SEM. The large numbers of plasmodesmata in the C_4_ species were still clustered but clearly distinct pit fields were difficult to identify (Fig. 2a–c; Videos [Link], [Link]). By contrast, plasmodesmata occurred in relatively few distinct pit fields in the C_3_ species.

**Fig. 3:**
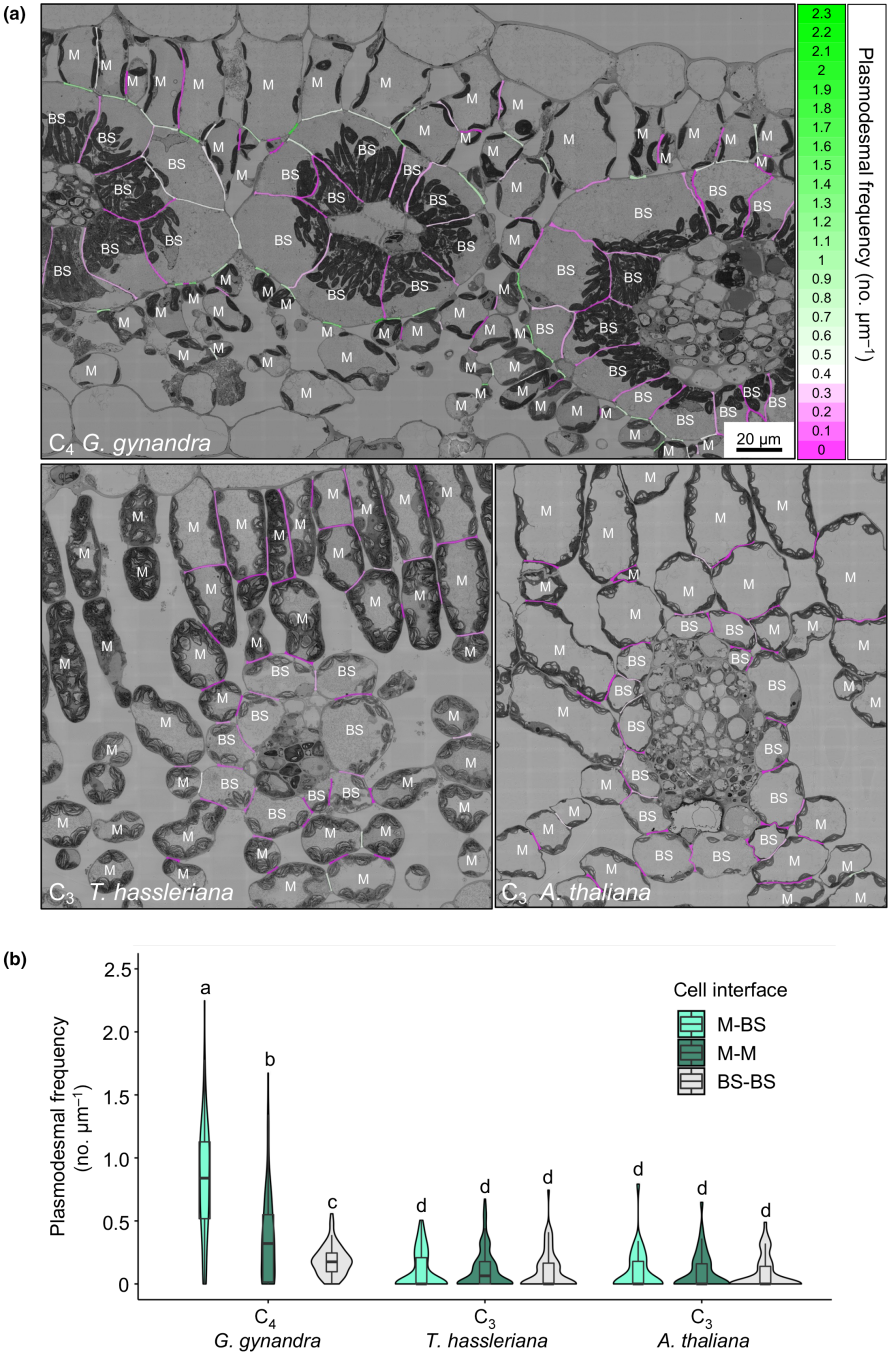
Plasmodesmata frequency in C_4_ *Gynandropsis gynandra* is higher between mesophyll (M) and bundle sheath (BS) cells compared with other interfaces. (a) Heatmap of plasmodesmata distribution. Cell interfaces in high‐resolution 2D SEM maps of C_4_ *G. gynandra*, C_3_ *Tarenaya hassleriana* and C_3_ *Arabidopsis thaliana* leaf cross‐sections were colored according to plasmodesmal frequency (number of plasmodesmata observed on the interface, divided by the interface length (μm)). Bars = 20 μm in all three images. (b) Plasmodesmal frequency for M–BS, M–M and BS–BS interfaces in *G. gynandra*, *T. hassleriana* and *A. thaliana* mature leaves quantified using high‐resolution 2D SEM maps. For *G. gynandra*, *n* = 86 (M–M), *n* = 96 (M–BS) and *n* = 70 (BS–BS) cell interfaces were quantified. For *T. hassleriana*, *n* = 202 (M–M), *n* = 80 (M–BS) and *n* = 77 (BS–BS) cell interfaces were quantified. For *A. thaliana*, *n* = 45 (M–M), *n* = 37 (M–BS) and *n* = 54 (BS–BS) cell interfaces were quantified. All interfaces were quantified from leaf samples of at least three individual plants (biological replicates) per species. Box and whiskers represent the 25 to 75 percentile and minimum–maximum distributions of the data. Letters show the statistical ranking using a *post hoc* Tukey test (different letters indicate statistically significant differences at *P* < 0.05). Values indicated by the same letter are not statistically different.

The SBF‐SEM provides an excellent 3D view of plasmodesmata frequency and distribution but is relatively low throughput and so limited numbers of cell interfaces can be visualized per unit time. We therefore used 2D electron microscopy to further explore the high occurrence of plasmodesmata at the M–BS cell interface of C_4_ *G. gynandra*. Large areas of leaf sections were imaged at high resolution using 2D SEM mapping such that automated serial imaging at 10 000× magnification and subsequent image stitching enabled visualization of plasmodesmata at numerous interfaces of the same 2D section (Fig. 3a). Representative SEM maps in which cell interfaces (M–BS, M–M and BS–BS) were pseudocolored according to plasmodesmal frequency, and consistent with the 3D SBF‐SEM analysis reported above, illustrated that plasmodesmata were specifically enriched at the M–BS interface of C_4_ *G. gynandra* (indicated by the numerous green‐colored interfaces). By contrast, frequency was lower and more uniform between all cellular interfaces in the C_3_ species (indicated by the pink and white pseudocolored cell interfaces; Fig. 3a). Plasmodesmata frequencies were quantified from at least three SEM maps originating from three independent plants (biological replicates; 10–40 individual M–BS, M–M and BS–BS cell interfaces per biological replicate). This showed that plasmodesmata numbers between M and BS cells were more than 8‐fold higher in C_4_ *G. gynandra* compared with both C_3_ species. The three cellular interfaces (M–BS, M–M and BS–BS) in both C_3_ species had similar plasmodesmal frequencies. Interestingly, plasmodesmal frequency of all three types of cell interface in *G. gynandra* was significantly higher than that of the corresponding interface in each of the two C_3_ species. For example, the M–M and BS–BS interfaces were approximately three‐ to fourfold and twofold higher in C_4_ *G. gynandra* compared with *T. hassleriana* and *A. thaliana* respectively indicating that cell‐to‐cell connectivity is generally enhanced between photosynthetic cells of the C_4_ species (Fig. 3b). Plasmodesmata frequencies estimated from analysis of numerous M–BS cell interfaces using this 2D SEM mapping were not statistically different from the frequencies obtained from multiple serial sections of the M–BS interface using SBF‐SEM (Fig. S2). To allow quantification of plasmodesmata in more diverse cell interfaces at greater replication and sampling, subsequent analysis was therefore carried out with the 2D SEM mapping technique.

### Increased plasmodesmal frequency between mesophyll and bundle sheath cells of C_4_ *G. gynandra* is established after exposure to light

**Fig. 4:**
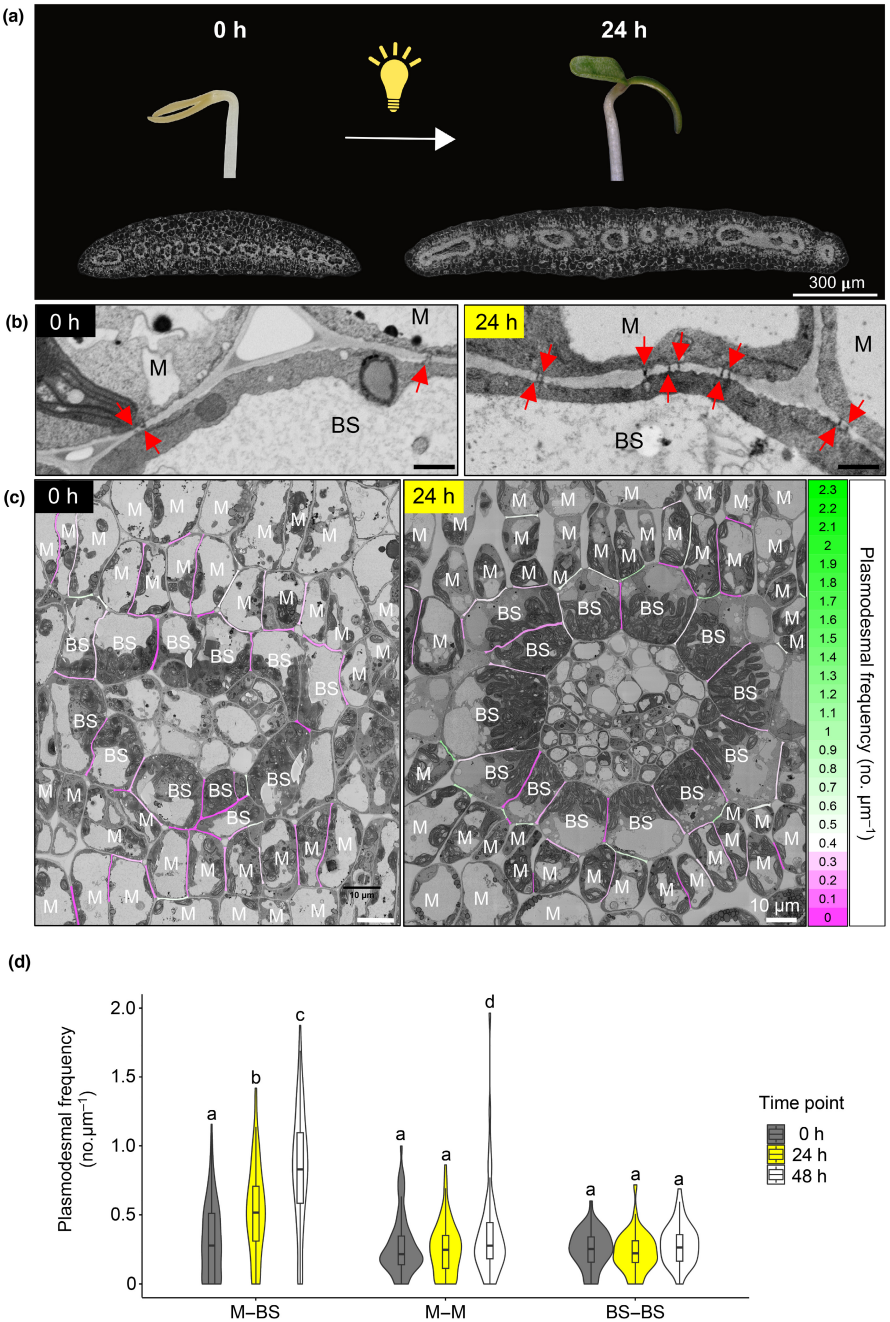
Light acts as a developmental cue to increase plasmodesmata formation at the mesophyll (M) and bundle sheath (BS) cell interface in cotyledons of C_4_ *Gynandropsis gynandra*. (a) Photographs of representative etiolated (left) and de‐etiolated (right) *G. gynandra* seedlings and scanning electron micrographs of cotyledon cross‐sections at 0 and 24 h after light. (b) Representative scanning electron micrographs of M–BS interfaces in C_4_ *G. gynandra* cotyledons. Red arrows indicate individual plasmodesma. Bar, 1 μm. (c) Heatmap of plasmodesmata distribution. Cell interfaces in high‐resolution 2D‐SEM maps of C_4_ *G. gynandra* cotyledon cross‐sections, harvested before light induction (0 h time point) and after light (24 h time point) were colored according to plasmodesmal frequency (number of plasmodesmata observed on the interface, divided by the interface length (μm)). (d) Plasmodesmata frequency per μm cell interfaces (M–BS, M–M, BS–BS) in *G. gynandra* cotyledons was quantified during dark‐to‐light transition (0, 24 and 48 h time points) using high‐resolution 2D SEM maps. For the 0 h time point, *n* = 81 (M–BS), *n* = 74 (M–M) and *n* = 49 (BS–BS) cell interfaces were quantified. For the 24 h time point, *n* = 69 (M–BS), *n* = 70 (M–M) and *n* = 42 (BS–BS) cell interfaces were quantified. For the 48 h time point, *n* = 90 (M–BS), *n* = 60 (M–M) and *n* = 49 (BS–BS) cell interfaces were quantified. All interfaces were quantified from cotyledon samples of at least 3 individual seedlings (biological replicates) per time point. The box and whiskers represent the 25 to 75 percentile and minimum–maximum distributions of the data. Letters show the statistical ranking using a one‐way ANOVA with a *post hoc* Tukey test (different letters indicate statistically significant differences at *P* < 0.05). Values indicated by the same letter are not statistically different.

Induction of the photosynthetic apparatus associated with the C_4_ pathway, such as chloroplast development and C_4_ gene expression, typically occurs rapidly in response to light (Shen *et al*., 2009; Singh *et al*., 2023). Such de‐etiolation analysis is simplest if cotyledons can be analyzed. As cotyledons of *G. gynandra* have C_4_ anatomy (Koteyeva *et al*., 2011), we examined plasmodesmata in this tissue during de‐etiolation. Cross‐sections of cotyledons showed that Kranz anatomy was already partially developed in 3‐d‐old dark‐grown seedlings (Fig. 4a). For example, veins were closely spaced, and BS cells contained abundant organelles. However, after 24 h of light cotyledons had almost doubled in size and substantial cell expansion and formation of air spaces were evident (Fig. 4c). High‐resolution 2D SEM maps from cross‐sections of at least three cotyledons (biological replicates) of *G. gynandra* were obtained at 0, 24 and 48 h after transfer to light. In dark‐grown seedlings, plasmodesmal frequency at M–BS, M–M and BS–BS were similar (*n* = 204; Fig. 4c,d). However, after light induction plasmodesmal frequency increased 1.7‐fold after 24 h and 2.5‐fold after 48 h between M and BS cells of *G. gynandra* (Fig. 4b–d). There was also a small increase in plasmodesmata numbers between M cells after light exposure. These responses were specific to de‐etiolation because growth in the dark for 48 h did not increase plasmodesmata numbers (Fig. S3a–d). These data indicate that as with true leaves, cotyledons of *G. gynandra* develop high plasmodesmal connectivity between M and BS cells, and that this takes place rapidly in response to light. We conclude that light is a crucial developmental cue for the formation of secondary plasmodesmata at the M–BS interface in the C_4_ plant *G. gynandra*.

### Functional chloroplasts are required for light‐induced formation of plasmodesmata between the mesophyll and bundle sheath

**Fig. 5:**
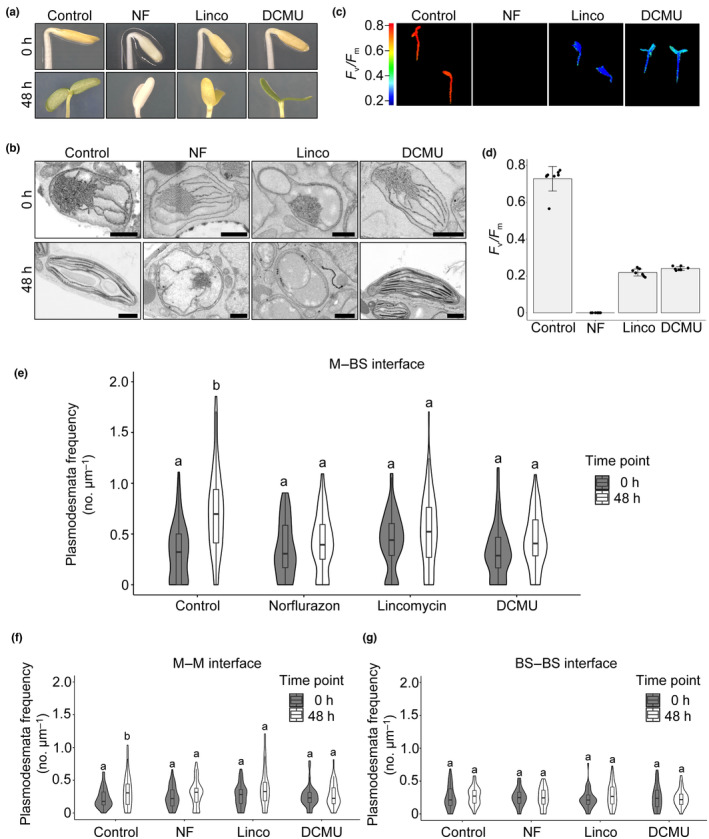
Inhibitors of chloroplast function reduce plasmodesmata formation at the mesophyll (M) and bundle sheath (BS) cell interface. The effect of norflurazon (NF), lincomycin (Linco) and 3‐(3,4‐dichlorophenyl)‐1,1‐dimethylurea (DCMU) were tested. (a) Photographs of *Gynandropsis gynandra* seedlings treated with inhibitors during de‐etiolation at 0 and 48 h. (b) Scanning electron micrographs of M etioplasts (0 h) and mature chloroplasts (48 h) in inhibitor‐treated and untreated (Control) *G. gynandra* seedlings. Bars, 1 μm. (c) Chlorophyll fluorescence images of maximum quantum efficiency of PSII photochemistry (*F*_v_/*F*_m_) from 48 h de‐etiolated *G. gynandra* seedlings treated with NF, Linco and DCMU, as well as untreated seedlings (Control). (d) *F*_v_/*F*_m_ measured in inhibitor‐treated and untreated *G. gynandra* seedlings at 48 h after light induction. Bars represent mean ± SD from *n* = 7–8 individual seedlings, dots represent individual data points. (e–g) Plasmodesmata frequency per μm cell interfaces in *G. gynandra* cotyledons was quantified during dark‐to‐light transition (0 and 48 h time point) for each individual inhibitor treatment using high‐resolution 2D SEM maps: (e) M–BS, (f) M–M and (g) BS–BS. (e) For M–BS interface: 0 h control *n* = 59, 0 h norflurazon *n* = 53, 0 h lincomycin *n* = 55, 0 h DCMU *n* = 50, 48 h control *n* = 85, 48 h norflurazon *n* = 66, 48 h lincomycin *n* = 90, 48 h DCMU *n* = 50 cell interfaces were quantified. (f) For M–M interface: 0 h control *n* = 41, 0 h norflurazon *n* = 43, 0 h lincomycin *n* = 45, 0 h DCMU *n* = 41, 48 h control *n* = 45, 48 h norflurazon *n* = 45, 48 h lincomycin *n* = 45, 48 h DCMU *n* = 45 cell interfaces were quantified. (g) For BS–BS interface: 0 h control *n* = 41, 0 h norflurazon *n* = 39, 0 h lincomycin *n* = 44, 0 h DCMU *n* = 38, 48 h control *n* = 45, 48 h norflurazon *n* = 45, 48 h lincomycin *n* = 43, 48 h DCMU *n* = 45 cell interfaces were quantified. All interfaces were quantified from cotyledon samples of at least three individual seedlings (biological replicates) per time point. The box and whiskers represent the 25–75 percentile and minimum–maximum distributions of the data. Letters show the statistical ranking, pairwise comparison of 0 and 48 h time point for each treatment, using a *post hoc* Tukey test (different letters indicate statistically significant differences at *P* < 0.05). Values indicated by the same letter are not statistically different.

De‐etiolation involves the transition from skotomorphogenic to photomorphogenic growth whereby fully photosynthetic chloroplasts develop from etioplasts within hours of light exposure (Cackett *et al*., 2021; Pipitone *et al*., 2021; Singh *et al*., 2023). Therefore, it is possible that the increase in plasmodesmal connectivity between M and BS cells during de‐etiolation is either a direct response to light or is triggered by signals from the chloroplast or photosynthesis. To investigate this, we used inhibitors with distinct modes of action to perturb chloroplast development and function. Lincomycin and norflurazon block plastid translation and carotenoid biosynthesis respectively and so stop the development of chloroplasts from etioplasts (Chamovitz *et al*., 1991; Mulo *et al*., 2003; Fig. 5b). 3‐(3,4‐dichlorophenyl)‐1,1‐dimethylurea (DCMU) blocks the electron transport chain at Photosystem II (PSII; Trebst, 2007) and thus inhibits photosynthesis directly. Seedlings were grown with and without each inhibitor and transferred to light for 48 h. Lincomycin‐ and DCMU‐treated seedlings had pale yellow cotyledons indistinguishable from nontreated controls. Norflurazon treatment generated seedlings with white cotyledons, consistent with compromised carotenoid accumulation (Fig. 5a). Etioplast ultrastructure was largely unaffected by the inhibitors except for the Lincomycin‐treated etioplasts that showed fewer prothylakoid structures deriving from the prolamellar body compared with untreated and DCMU‐ and NF‐treated *G. gynandra* seedlings (Fig. 5b). After 48 h of light, cotyledons of controls and DCMU‐treated seedlings were green and etioplasts had developed into chloroplasts (Fig. 5a,b). Norflurazon and lincomycin‐treated seedlings had pale cotyledons even after light induction and the etioplast‐to‐chloroplast development was arrested (Fig. 5a,b). To confirm that each inhibitor had the expected effect on chloroplast function, we used chlorophyll fluorescence imaging to quantify *F*_v_/*F*_m_ which provides a read‐out for the maximum quantum efficiency of Photosystem II. Each inhibitor drastically reduced *F*_v_/*F*_m_ compared with controls (Fig. 5c,d). Norflurazon‐treated seedlings were not visible on the chlorophyll fluorescence imager as chlorophyll content was too low.

Using 2D SEM maps, we quantified plasmodesmal frequency in *c*. 50 cell interfaces for each treatment and each time point. None of the three inhibitors affected plasmodesmal frequency at any cell interface in dark‐grown seedlings (Fig. 5e–g). However, despite cotyledon expansion being unaffected by the inhibitors during de‐etiolation (Fig. S4a), plasmodesmal frequencies did not increase significantly in seedlings treated with norflurazon, lincomycin or DCMU (Figs 5e–g, S4b). In summary, inhibitors that perturbed the etioplast‐to‐chloroplast transition or blocked photosynthetic electron transport reduced light‐induced plasmodesmata formation between M and BS cells of C_4_ *G. gynandra*. We conclude that chloroplast function, and in particular photosynthetic electron transport, play important roles in controlling the formation of secondary plasmodesmata in the C_4_ leaf.

**Fig. 6:**
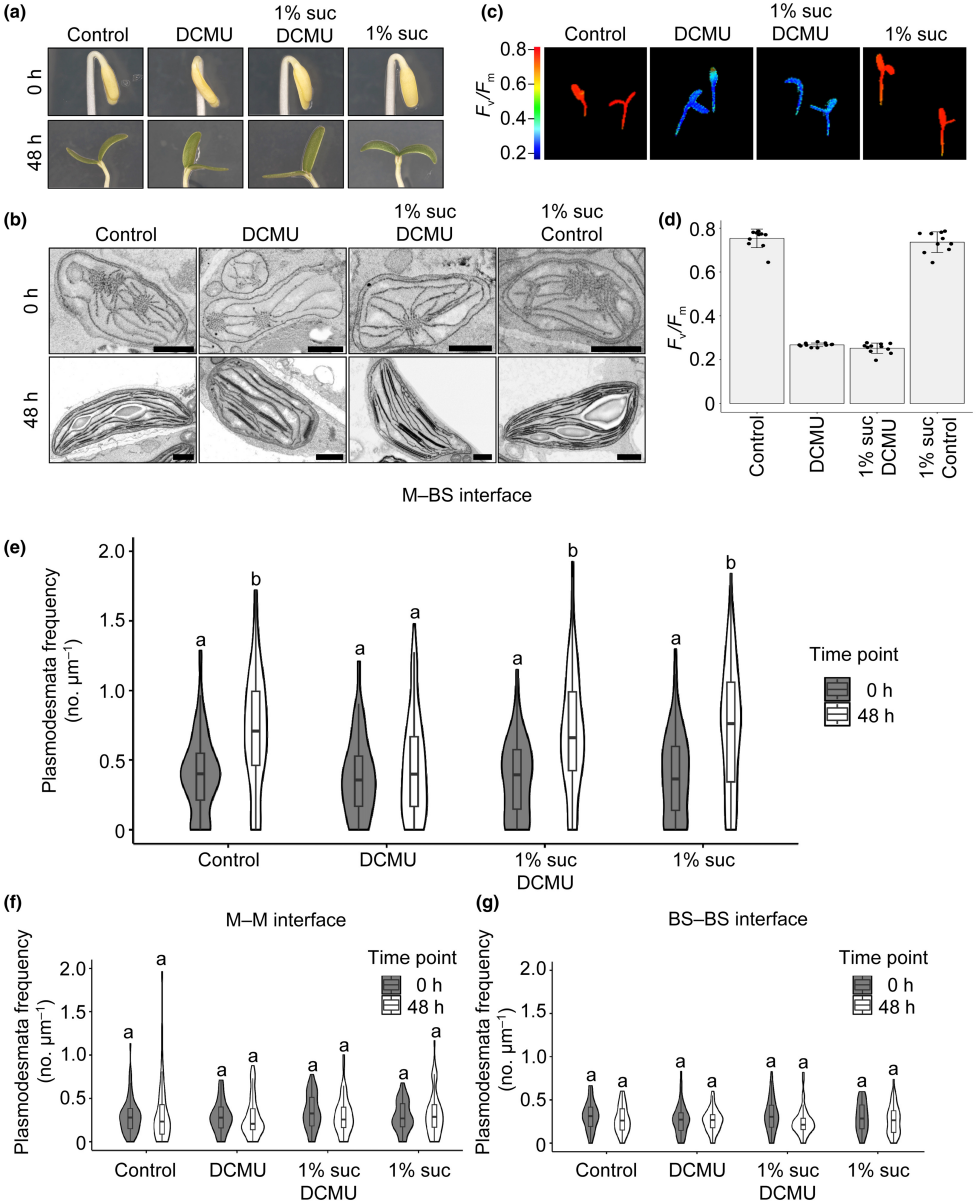
Sucrose rescues 3‐(3,4‐dichlorophenyl)‐1,1‐dimethylurea (DCMU)‐mediated inhibition of plasmodesmata formation at the mesophyll (M) and bundle sheath (BS) cell interface. (a) Representative images of DCMU‐treated *Gynandropsis gynandra* seedlings during de‐etiolation (at 0 h and 48 h) with or without exogenous 1% (w/v) sucrose. (b) Scanning electron micrographs of M etioplasts (0 h) and mature chloroplasts (48 h) of DCMU‐treated and untreated (Control) *G. gynandra* seedlings with or without exogenous 1% (w/v) sucrose. Bars, 1 μm. (c) Chlorophyll fluorescence images of maximum quantum efficiency of PSII photochemistry (*F*_v_/*F*_m_) from 48 h de‐etiolated, untreated and DCMU‐treated seedlings. (d) *F*_v_/*F*_m_ measured in *G. gynandra* 48 h after light induction. Bars represent mean ± SD from *n* = 7–8 individual seedlings, dots represent individual data points. (e–g) Plasmodesmata frequency per μm cell interfaces in *G. gynandra* cotyledons quantified during the dark‐to‐light transition (0 and 48 h time point) and DCMU treatment, with and without additional 1% (w/v) sucrose supply using high‐resolution 2D SEM maps: (e) M–BS, (f) M–M and (g) BS–BS. All interfaces were quantified from cotyledon samples of at least three individual seedlings (biological replicates) per time point. (e) For M–BS interface: 0 h control *n* = 96, 0 h DCMU *n* = 82, 0 h 1% suc *n* = 84, 0 h 1% suc DCMU *n* = 87, 48 h control *n* = 79, 48 h DCMU *n* = 98, 48 h 1% suc *n* = 101, 48 h 1% suc DCMU *n* = 96 cell interfaces were quantified. (f) For M–M interface: 0 h control *n* = 64, 0 h DCMU *n* = 57, 0 h 1% suc *n* = 65, 0 h 1% suc DCMU *n* = 63, 48 h control *n* = 55, 48 h DCMU *n* = 60, 48 h 1% suc *n* = 58, 48 h 1% suc DCMU *n* = 55 cell interfaces were quantified. (g) For BS–BS interface: 0 h control *n* = 65, 0 h DCMU *n* = 62, 0 h 1% suc *n* = 53, 0 h 1% suc *n* = 57, 48 h control *n* = 48, 48 h DCMU *n* = 53, 48 h 1% suc *n* = 62, 48 h 1% suc DCMU *n* = 55 cell interfaces were quantified. The box and whiskers represent the 25–75 percentile and minimum–maximum distributions of the data. Letters show the statistical ranking, pairwise comparison of 0 and 48 h time point for each treatment, using a *post hoc* Tukey test (different letters indicate statistically significant differences at *P* < 0.05). Values indicated by the same letter are not statistically different.

The inhibitory effect of DCMU on plasmodesmata formation could be associated with signaling from a dysfunctional photosynthetic electron transport chain, or because less photosynthate is produced. To test the latter, hypothesis plants were grown on sucrose during DCMU treatment. No distinguishable effects on phenotype of the seedlings or etioplast‐to‐chloroplast development were detected (Fig. 6a,b) and provision of sucrose did not rescue the reduction in *F*_v_/*F*_m_ caused by DCMU (Fig. 6c,d). However, when we quantified plasmodesmal frequencies, DCMU‐treated seedlings supplemented with sucrose had plasmodesmal frequencies at the M–BS interface that were comparable to those in untreated seedlings (Fig. 6e, *P* > 0.05) indicating full rescue by sucrose of the DCMU‐induced inhibition of plasmodesmata formation. Thus, when photosynthetic electron transport is inhibited, sucrose is sufficient to restore plasmodesmata formation at the M–BS cell interface of *G. gynandra*. However, we found that the exogenous supply of sucrose did not rescue norfluorazon‐mediated inhibition of plasmodesmata formation during light induction (Fig. S5). In contrast with DCMU treatment where chloroplasts developed normally, norflurazon completely abolished the development of chloroplasts (Fig. 5a). We therefore propose that functional chloroplasts are required as sucrose is not sufficient to fuel plasmodesmata formation in plants with nonfunctional chloroplasts (e.g. in norflurazon‐treated seedlings).

**Fig. 7:**
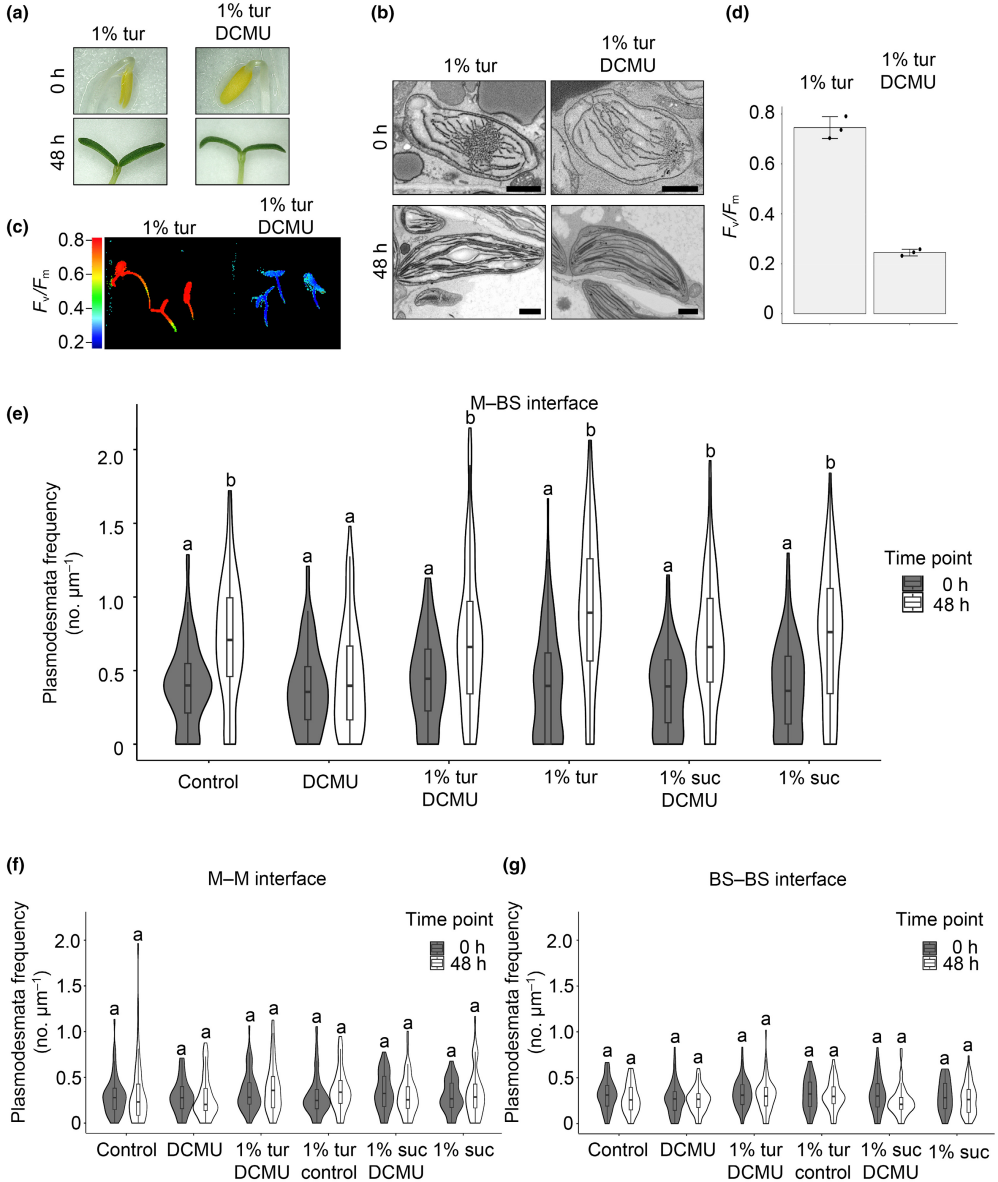
Nonmetabolizable sucrose analog (turanose) can partially rescue 3‐(3,4‐dichlorophenyl)‐1,1‐dimethylurea (DCMU)‐mediated inhibition of plasmodesmata formation at the mesophyll (M) and bundle sheath (BS) cell interface. (a) Representative images of DCMU‐treated and untreated *Gynandropsis gynandra* seedlings during de‐etiolation (at 0 h and 48 h) with exogenous 1% (w/v) turanose (tur). (b) Scanning electron micrographs of M etioplasts (0 h) and mature chloroplasts (48 h) of DCMU‐treated and untreated *G. gynandra* seedlings with exogenous 1% (w/v) turanose. Bars, 1 μm. (c) Chlorophyll fluorescence images of maximum quantum efficiency of PSII photochemistry (*F*_v_/*F*_m_) from 48 h de‐etiolated, untreated and DCMU‐treated seedlings grown with exogenous 1% (w/v) turanose (tur). (d) *F*_v_/*F*_m_ measured in *G. gynandra* 48 h after light induction. Scale bars represent mean ± SD from *n* = 3 individual seedlings; dots represent individual data points. (e–g) Plasmodesmata frequency per μm cell interfaces in *G. gynandra* cotyledons quantified during the dark‐to‐light transition (0 and 48 h time point) and DCMU treatment, with and without additional 1% (w/v) sucrose or 1% (w/v) turanose supply using high‐resolution 2D SEM maps: (c) M–M, (d) M–BS and (e) BS–BS. All interfaces were quantified from cotyledon samples of at least 3 individual seedlings (biological replicates) per time point. Data for untreated and DCMU‐treated samples grown with and without exogenous 1% sucrose were replotted from Fig. 6(e–g). (e) For M–BS interface: 0 h 1% turanose DCMU *n* = 91, 0 h 1% turanose *n* = 101, 48 h 1% turanose DCMU *n* = 159, 48 h 1% turanose *n* = 91 cell interfaces were quantified. (f) For M–M interface: 0 h 1% turanose DCMU *n* = 62, 0 h 1% turanose *n* = 75, 48 h 1% turanose DCMU *n* = 101, 48 h 1% turanose *n* = 61 cell interfaces were quantified. (g) For BS–BS interface: 0 h 1% turanose DCMU *n* = 60, 0 h 1% turanose *n* = 60, 48 h 1% turanose DCMU *n* = 88, 48 h 1% turanose *n* = 52 cell interfaces were quantified. The box and whiskers represent the 25–75 percentile and minimum–maximum distributions of the data. Letters show the statistical ranking, pairwise comparison of 0 and 48 h time point for each treatment, using a *post hoc* Tukey test (different letters indicate statistically significant differences at *P* < 0.05). Values indicated by the same letter are not statistically different.

We further tested whether sucrose could act as a metabolite or signaling molecule to induce secondary plasmodesmata formation during de‐etiolation. To do this, we grew DCMU‐treated *G. gynandra* seedlings on turanose – a nonmetabolizable sucrose analog. Again, there were no distinguishable effects on seedling phenotypes or etioplast‐to‐chloroplast development (Fig. 7a,b) and provision of turanose did not affect the reduction in *F*_v_/*F*_m_ caused by DCMU (Fig. 7c). Plasmodesmal frequencies at the M–BS interface after 48 h of light induction were slightly lower than those in untreated seedlings (Fig. 7d–g), indicating a partial rescue by turanose of the DCMU‐induced inhibition of plasmodesmata formation. These results indicate that sugar signaling likely contributes to plasmodesmata formation at the M–BS cell interface of *G. gynandra*.

## Discussion

### Increased plasmodesmata frequency is a conserved C_4_ trait

A critical feature of the C_4_ pathway is the spatial separation of biochemical processes such that CO_2_ can be concentrated around RuBisCO. The consequence of this partitioning of photosynthesis is an absolute requirement for the exchange of metabolites between cell types. In C_4_ grasses, this has long been associated with increased plasmodesmal frequency between M and BS cells (Evert *et al*., 1977). Previous work quantified plasmodesmata frequency at the M–BS cell interface of *G. gynandra* and yielded comparable values for plasmodesmata frequencies as in our work (Koteyeva *et al*., 2014), but they did not quantify plasmodesmata in any other cell interface or compared plasmodesmal frequency with related C_3_ species. Therefore, despite the very different leaf morphology between monocotyledons and dicotyledons, our results reveal that increased plasmodesmal connectivity between M and BS cells is likely a conserved trait among C_4_ plants that separate photosynthesis between two cell types. In *G. gynandra*, the M–BS interfaces had 8‐ to 13‐fold higher plasmodesmata frequency than those of the closely related C_3_ species *T. hassleriana* and *A. thaliana* (Figs 1, 2, 3). This increase is comparable to plasmodesmata numbers and distributions reported for C_4_ grasses (Botha, 1992; Danila *et al*., 2016). Danila *et al*. (2018) further reported that C_4_ grasses running the NAD‐ME subtype of C_4_ photosynthesis had the highest numbers of plasmodesmata between M and BS cells. As *G. gynandra* also primarily uses NAD‐ME to decarboxylate CO_2_ in the BS, broader analysis of C_4_ dicotyledons is required to determine the extent to which plasmodesmal frequencies correlate with the various biochemical subtypes.

Plasmodesmal frequencies at the M–BS interface of *G. gynandra* are consistent with those reported previously in this species where no analysis of closely related C_3_ plants was possible (Koteyeva *et al*., 2014). By quantifying plasmodesmata at all interface types and comparing plasmodesmal frequency with phylogenetically proximate C_3_ plants, we demonstrate that plasmodesmata numbers are generally higher at all three types of cell interface (M–BS, M–M and BS–BS) in C_4_ *G. gynandra*. This is consistent with previous work that observed increased plasmodesmata frequencies between photosynthetic leaf cells in C_4_ grasses compared with C_3_ grasses (Danila *et al*., 2016).

In contrast with previous work on C_4_ grasses and the C_3_ species that we studied here (*T. hassleriana* or *A. thaliana*), due to the very large numbers of plasmodesmata, we were not able to distinguish clear pit fields between M and BS cells of *G. gynandra* (Fig. 2a–c; Videos [Link], [Link]). This might suggest that the mechanism for increased plasmodesmata numbers in *G. gynandra* is an increase in pit fields and/or an increase in pit field area to the extent that plasmodesmata no longer appear in clusters and are rather evenly distributed – potentially ensuring an uniform metabolic flux of C_4_ acids across the cell interface. How this phenomenon relates to that seen in C_4_ grasses, where the increased plasmodesmal frequency was accompanied by increases in pit field area such that they were up to five times greater than those in C_3_ species (Danila *et al*., 2016, 2018), requires further investigation. It is therefore possible that mechanisms underpinning increased plasmodesmata numbers between M and BS cells vary between C_4_ lineages, and might be distinct between cell walls with and without suberin deposition.

Flux of metabolites between cells is likely to be determined by plasmodesmata number as increased numbers can facilitate greater flux (in addition to their regulation; Turgeon & Medville, 2004; Amiard *et al*., 2005; Danila *et al*., 2016). However, BS cells are not air‐tight and plasmodesmata could also contribute to CO_2_ leakiness such that a proportion of the CO_2_ concentrated in the BS diffuses back to the M. CO_2_ leakiness particularly increases during photosynthetic induction in NADP‐ME type C_4_ plants such as sorghum and maize (Wang *et al*., 2022). Thus, it is possible that plasmodesmata number and distribution need to be optimized to allow maximum photosynthetic efficiency in C_4_ plants during leaf development. Being able to accurately quantify plasmodesmal traits in diverse C_4_ species may be crucial to developing further understanding in this area, and in particular in modelling metabolite flux through the C_4_ pathway (Danila *et al*., 2016; Von Caemmerer, 2021). This could incorporate recent models of metabolite diffusion through plasmodesmata such as the geometric and narrow escape models (Deinum *et al*., 2019; Hughes *et al*., 2021).

### Light triggers rapid plasmodesmata formation in pre‐existing cell walls

In C_4_ grasses, the developmental cue that enhances plasmodesmata formation between M and BS cells is not known. However, *Setaria viridis* and maize show some plasticity in plasmodesmal density in response to growth irradiance (Danila *et al*., 2019). Our data provide a direct link between light and photosynthesis in establishing plasmodesmal frequency by showing that light rapidly triggers the formation of plasmodesmata at the M–BS interface in *G. gynandra*.

We believe that the increase in plasmodesmata numbers between M and BS cell during dark‐to‐light transition is primarily driven by the formation of secondary plasmodesmata for the following reasons. First, cotyledon growth from dark to light is thought to be exclusively driven by cell expansion and not cell division in Arabidopsis (Tsukaya *et al*., 1994; Stoynova‐Bakalova *et al*., 2004). Second, the basic structure of BS cells was already formed in dark‐grown seedlings, and the formation of plasmodesmata was rapid. Our SEM mapping technique provided sufficient resolution to observe branching in plasmodesmata (Figs 4, 5, 6), but interestingly, we did not observe any structural differences between the plasmodesmata at different cell interfaces. Plasmodesmata were mostly simple in structure and some X, Y or H‐shaped – typical for nonmature leaf tissues (Roberts *et al*., 2001). Primary and secondary plasmodesmata can be sometimes distinguished by structure, with the latter being more branched, but this is highly dependent on other factors such as leaf age and sink‐source transition (Roberts *et al*., 2001). Gao *et al*. (2022) found that plasmodesmata at the M to BS interface of maize acquire defined sphincters and cytoplasmic sleeves and this coincided with both dimorphic chloroplast development and BS cell wall suberization. However, these structural features were not observed during de‐etiolation, nor in fully mature leaves in *G. gynandra*.

### A role for metabolism and organelles in formation of plasmodesmata

Our results suggest that chloroplasts, and more specifically photosynthesis, fuel the formation of secondary plasmodesmata between M and BS cells in C_4_ *G. gynandra*. Inhibition of photochemical reactions of photosynthesis and chloroplast development through the application of inhibitors greatly reduced plasmodesmata formation during de‐etiolation but this effect could be rescued by the exogenous supply of sucrose (Figs 5 and 6). To our knowledge, a role of photosynthate in controlling formation of plasmodesmata has not been proposed previously. However, some findings are consistent with this hypothesis. For example, in rice constitutive overexpression of the C_4_ maize *GOLDEN2‐LIKE* transcription that controls chloroplast biogenesis (Waters *et al*., 2008) not only activated chloroplast and mitochondria development in BS cells but also increased plasmodesmata numbers (Wang *et al*., 2017). Moreover, in *A. thaliana* links between organelles and plasmodesmata have been reported. *A. thaliana* mutants with altered cell‐to‐cell connectivity and/or plasmodesmata structure such INCREASED SIZE EXCLUSION LIMIT1 and 2 (ISE1/ISE2) encode mitochondrial and chloroplast RNA helicases respectively (Kobayashi *et al*., 2007; Stonebloom *et al*., 2009), while the GFP ARRESTED TRAFFICKING1 (GAT1) locus encodes a chloroplast thioredoxin (Benitez‐Alfonso *et al*., 2009). However, the mechanisms of how these organelle‐localized proteins affect plasmodesmata formation are not understood. Retrograde signaling from chloroplast to nucleus has also been proposed to control plasmodesmata formation and regulation (Burch‐Smith *et al*., 2011; Ganusova *et al*., 2020). The fact that exogenous supply of sucrose is sufficient to sustain plasmodesmata formation in the presence of DCMU (Fig. 6) strongly suggests a direct metabolic role of chloroplasts in the enhanced formation of plasmodesmata in the C_4_ leaf. This may involve sucrose or photosynthesis providing energy required for plasmodesmata formation, or sucrose acting as a signaling molecule to trigger plasmodesmata formation. Consistent with the latter hypothesis, the nonmetabolizable sucrose analog turanose, partially rescued the DCMU‐inhibited plasmodesmata formation (Fig. 7). Further work will be required to identify the specific sugar signaling pathway involved. Interestingly, TARGET OF RAPAMYCIN (TOR) signaling was previously implicated in the control of plasmodesmata‐mediated cell‐to‐cell connectivity (Brunkard *et al*., 2020). Further work is also required to test whether the increase in plasmodesmal frequencies directly leads to an increase in metabolic flux of C_4_ metabolites.

In summary, our work demonstrates that increased plasmodesmal connectivity is likely a conserved trait found in both C_4_ dicotyledons and monocotyledons. Moreover, the enhanced formation of plasmodesmata between M and BS cells of C_4_ leaves is coordinated and dependent on photosynthesis. Evolution therefore appears to have wired the enhanced formation of plasmodesmata in C_4_ leaves to the development of chloroplasts and ultimately the induction of photosynthesis.

## Competing interests

None declared.

## Author contributions

TBS and JMH conceived and directed the research. TBS, CF, SCZ and JMH designed the experiments. TBS, KM and SE performed the research. TBS analyzed the data. TBS and JMH wrote the article with input from all the authors.

## Supporting information


**Fig. S1** Transmission electron micrographs of mesophyll (M)–bundle sheath (BS), M–M and BS–BS cell interfaces.
**Fig. S2** Comparison of plasmodesmal frequencies quantified at Mesophyll and bundle sheath cell interface using 2D SEM and 3D SBF‐SEM.
**Fig. S3** Extended dark treatment for 48 h does not increase plasmodesmata frequency in *Gynandropsis gynandra* cotyledons.
**Fig. S4** Chloroplast inhibitors have limited effect on light‐induced cotyledon expansion, but affect plasmodesmata formation.
**Fig. S5** Sucrose cannot rescue norfluorazon‐mediated inhibition of plasmodesmata formation at the M–BS interface.

**Video S1** 3D reconstruction video of mesophyll and bundle sheath cell interface in a mature *Gynandropsis gynandra* leaf.

**Video S2** 3D reconstruction video of mesophyll and bundle sheath cell interface in a mature *Tarenaya hassleriana* leaf.

**Video S3** 3D reconstruction video of mesophyll and bundle sheath cell interface in a mature *Arabidopsis thaliana* leaf.

**Video S4** Compiled video of sequential 50 nm sections of mesophyll and bundle sheath cell interface in mature leaves of C_4_ *Gynandropsis gynandra*.

**Video S5** Compiled video of sequential 50 nm sections of mesophyll and bundle sheath cell interface in mature leaves of C_3_ *Tarenaya hassleriana*.

**Video S6** Compiled video of sequential 50 nm sections of mesophyll and bundle sheath cell interface in mature leaves of C_3_ *Arabidopsis thaliana*.
Please note: Wiley is not responsible for the content or functionality of any Supporting Information supplied by the authors. Any queries (other than missing material) should be directed to the *New Phytologist* Central Office.

## Data Availability

The data that support the findings of this study are available in the main figures and Supporting Information.
