# Supplementary material for: Plasmodesmal connectivity in C4 Gynandropsis gynandra is induced by light and dependent on photosynthesis
Source: New Phytol. 2023 Oct 26;241(1):298–313. doi: 10.1111/nph.19343 (PMC10952754; doi:10.1111/nph.19343)
Supplement: Supplementary file 1 — Fig. S1 Transmission electron micrographs of mesophyll (M)–bundle sheath (BS), M–M and BS–BS cell interfaces. Fig. S2 Comparison of plasmodesmal frequencies quantified at Mesophyll and bundle sheath cell interface using 2D SEM and 3D SBF‐SEM. Fig. S3 Extended dark treatment for 48 h does not increase plasmodesmata frequency in Gynandropsis gynandra cotyledons. Fig. S4 Chloroplast inhibitors have limited effect on light‐induced cotyledon expansion, but affect plasmodesmata formation. Fig. S5 Sucrose cannot rescue norfluorazon‐mediated inhibition of plasmodesmata formation at the M–BS interface. [file NPH-241-298-s007.pdf]

New Phytologist Supporting Information

Article title: Plasmodesmal connectivity in C4 *Gynandropsis gynandra* is induced by light and dependent on photosynthesis

Authors: Tina B. Schreier, Karin H. Müller, Simona Eicke, Christine Faulkner, Samuel C. Zeeman and Julian M. Hibberd

Article acceptance date: 28 September 2023

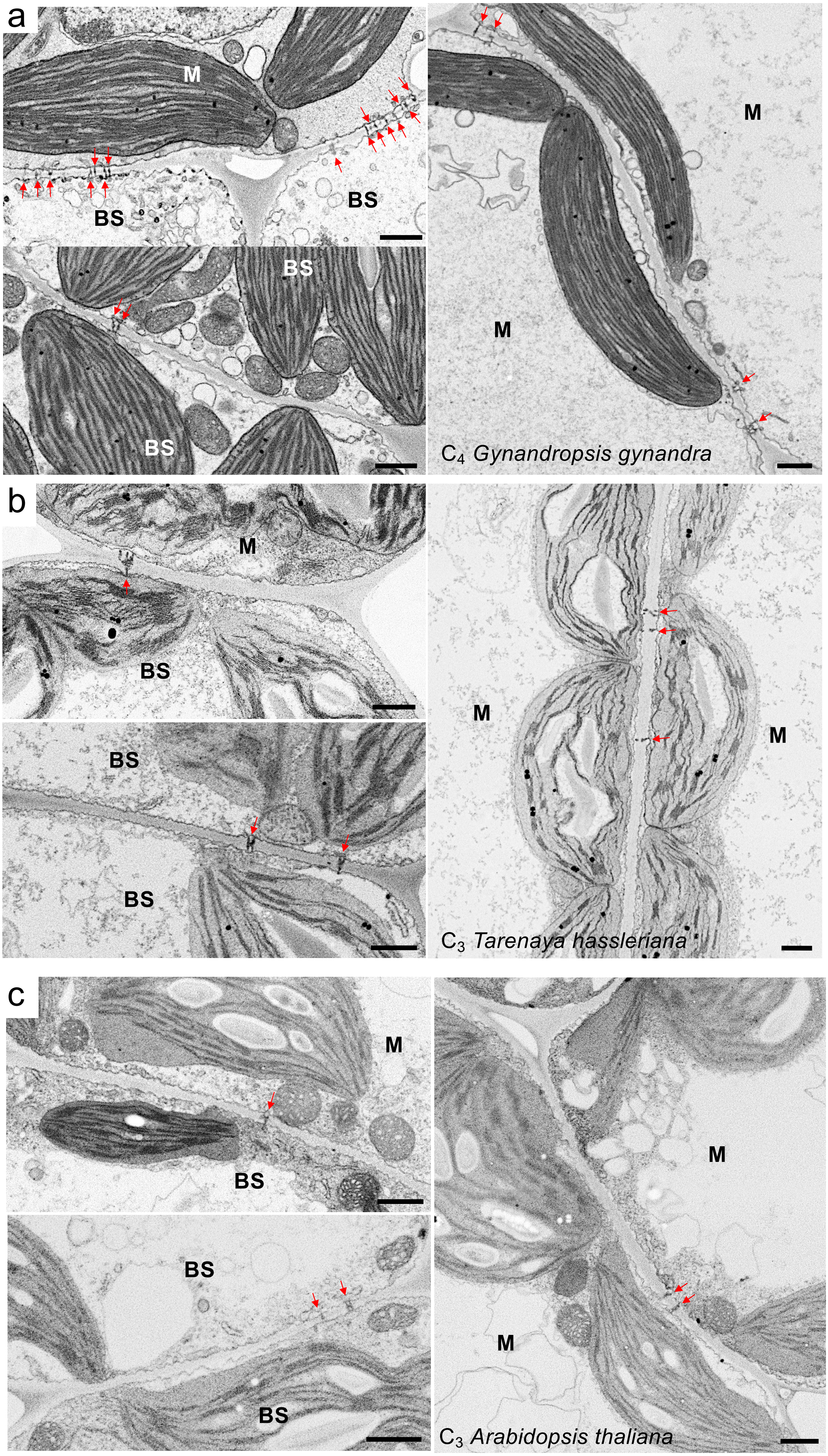
a
M
BS
BS
BS
BS
M
M
C_4 *Gynandropsis gynandra*
b
M
BS
BS
BS
M
M
C_3 *Tarenaya hassleriana*
c
M
BS
BS
BS
M
M
C_3 *Arabidopsis thaliana*

Supporting Information Figure S1. Transmission electron micrographs of Mesophyll (M) - Bundle Sheath (BS), M-M and BS-BS cell interfaces. Representative interfaces in (**a**) C_4 *G. gynandra*, (**b**) C_3 *T. hassleriana* and (**c**) C_3 *A. thaliana* leaves. Mature leaves were harvested from 4-week-old *G. gynandra* and *T. hassleriana* plants and 3-week-old *A. thaliana*. Red arrows indicate individual plasmodesma. Scale bar = 1 µm.

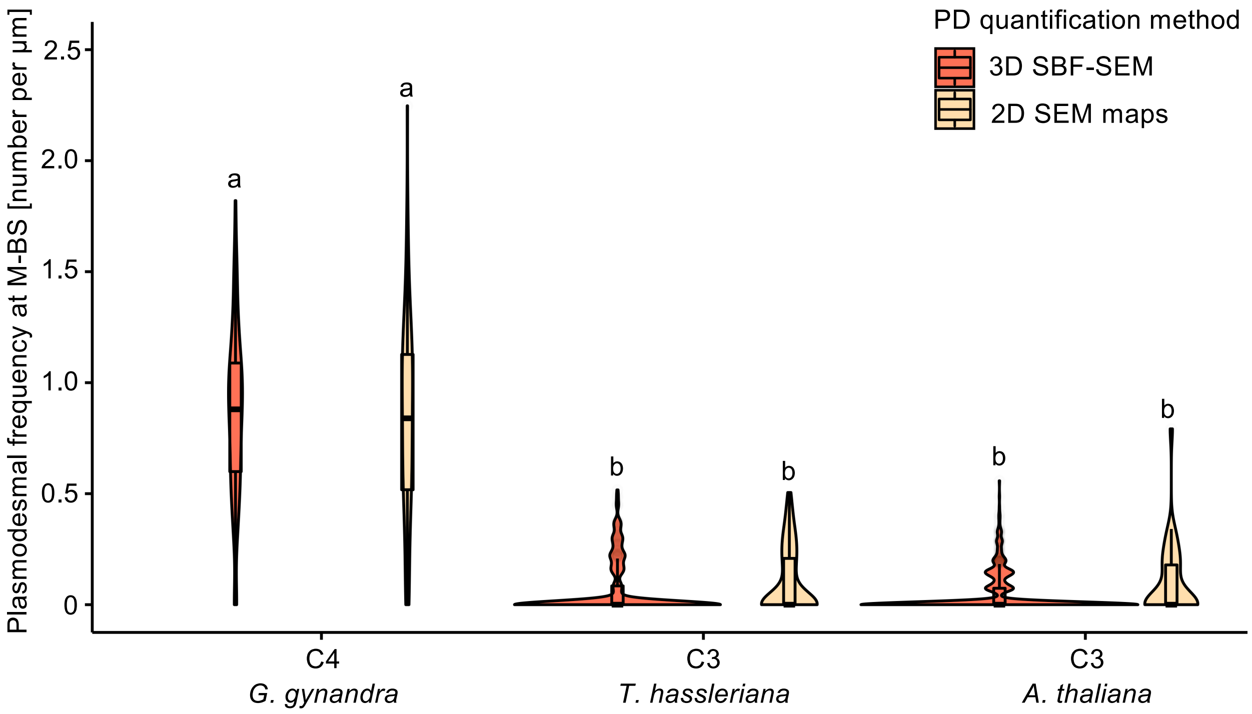

Supporting Information Figure S2. Comparison of plasmodesmal frequencies quantified at Mesophyll (M) and Bundle Sheath (BS) cell interface using 2D SEM and 3D SBF-SEM. Numerous M-BS cell interfaces were quantified in 2D SEM maps, while few M-BS cell interfaces were analysed in-depth using 3D SBF-SEM. The box and whiskers represent the 25 to 75 percentile and minimum-maximum distributions of the data. Letters show the statistical ranking using a *post hoc* Tukey test (different letters indicate significant differences at $P < 0.05$). Values indicated by the same letter are not statistically different.

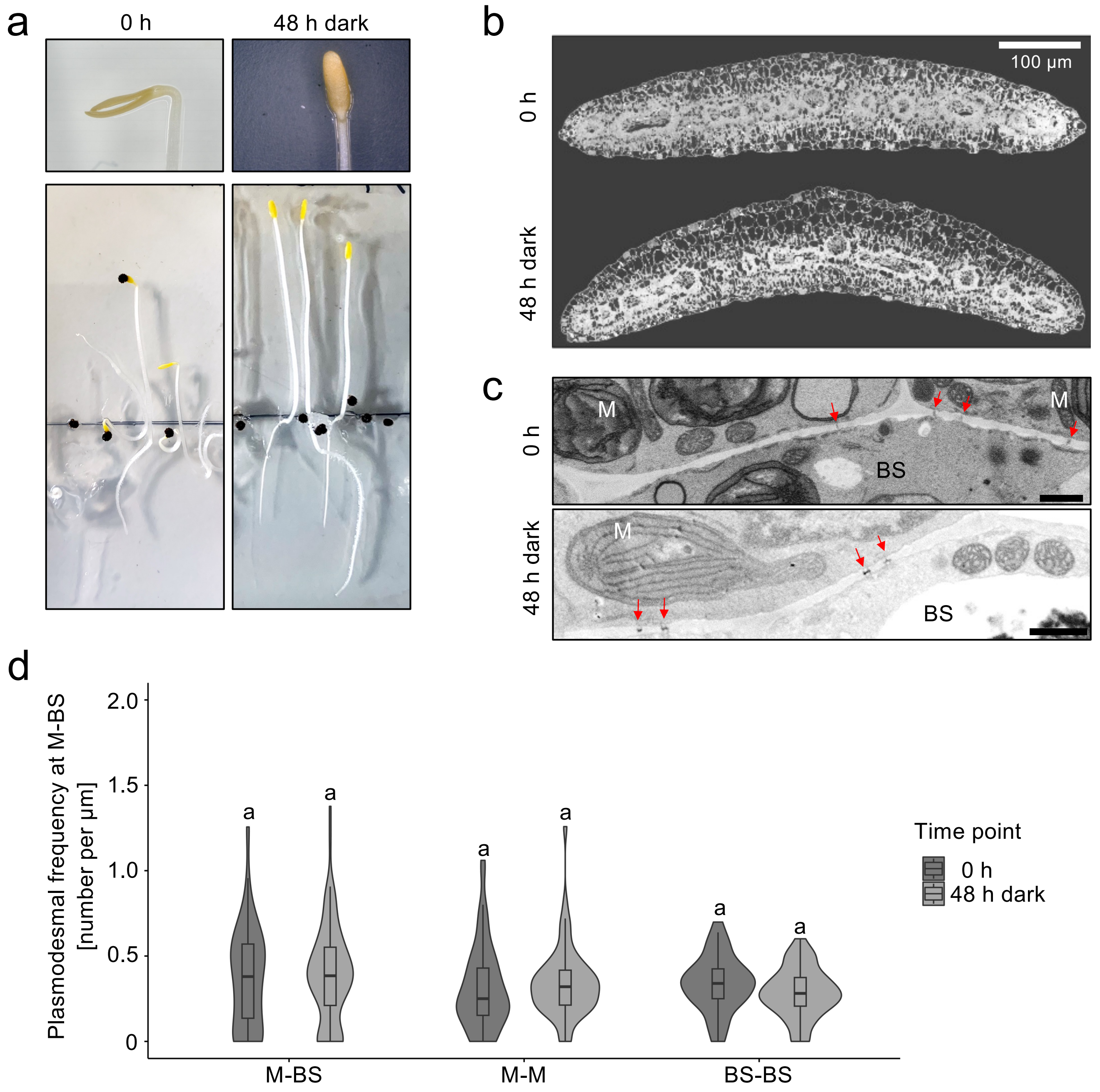

Supporting Information Figure S3. Extended dark treatment for 48 h does not increase plasmodesmata frequency in *G. gynandra* cotyledons. (**a**) Photographs of 3-day-old (0) and 5-day-old (48 h extended dark) dark-grown *G. gynandra* seedlings on half-strength MS media. (**b**) Scanning electron micrographs of entire cotyledon cross sections of 0 h and 48 h extended dark-treated *G. gynandra* seedlings. (**c**) Representative scanning electron micrographs of Mesophyll (M) and Bundle Sheath (BS) cell interfaces of 0 h and 48 h extended dark-treated *G. gynandra* cotyledons. Red arrows indicate individual plasmodesma. Scale bar = 1 µm (**d**) Plasmodesmal frequency per µm cell interfaces (for M-BS, M-M and BS-BS) in *G. gynandra* cotyledons was quantified before and after extended dark treatment (0 h and 48 h dark time point) using high-resolution 2D SEM maps. For the 0 h time point, *n* = 84 (M-BS), *n* = 60 (M-M), and *n* = 41 (BS-BS) cell interfaces were quantified. For the 48 h dark time point, *n* = 91 (M-BS), *n* = 60 (M-M), and *n* = 58 (BS-BS) cell interfaces were quantified. All interfaces were quantified from cotyledon samples of at least 3 individual seedlings (biological replicates) per time point. The box and whiskers represent the 25 to 75 percentile and minimum-maximum distributions of the data. Letters show the statistical ranking using a *post hoc* Tukey test (different letters indicate significant differences at P<0.05). Values indicated by the same letter are not statistically different.

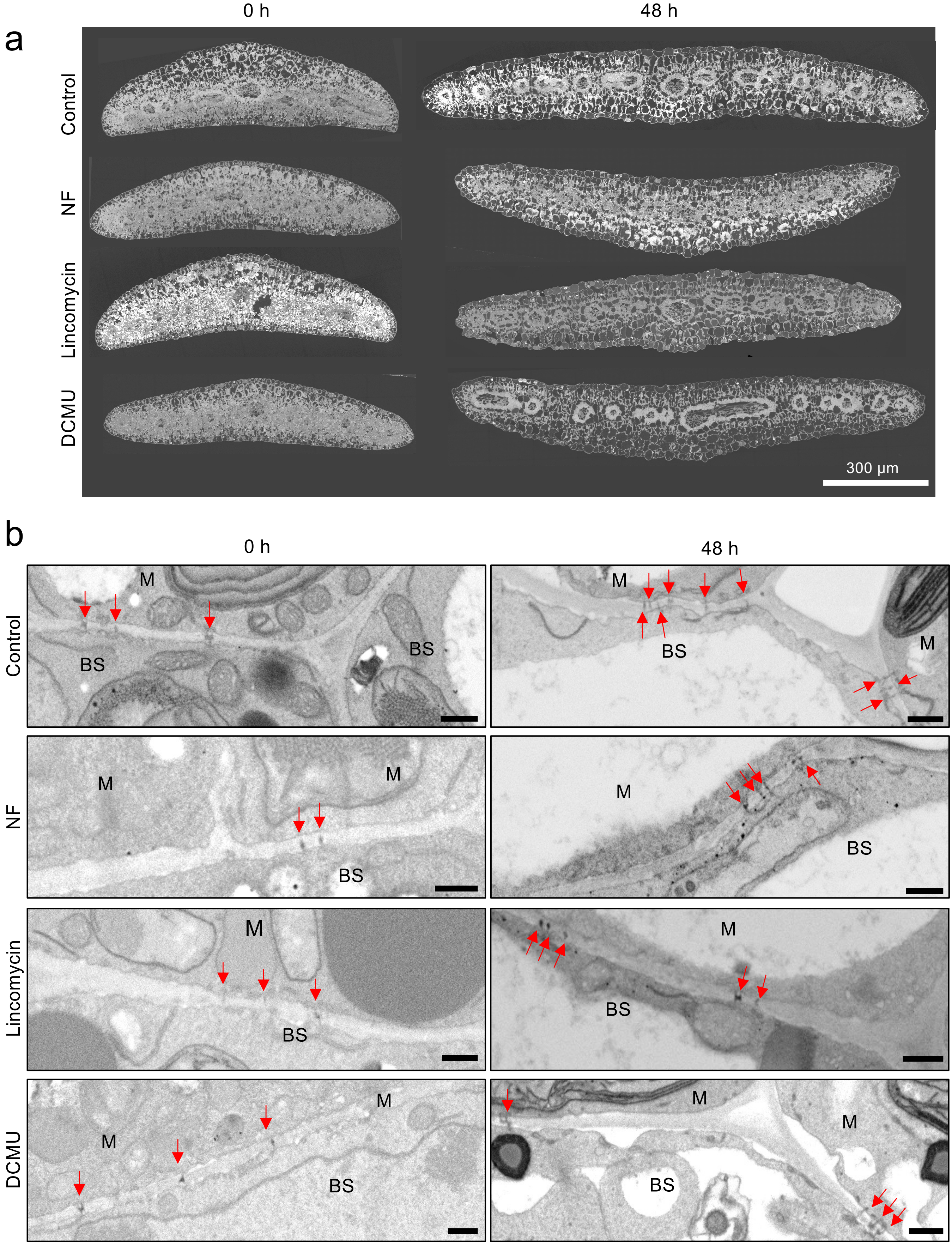
a
0 h
48 h
Control
NF
Lincomycin
DCMU
300 μm
b
0 h
48 h
Control
NF
Lincomycin
DCMU
M
BS

Supporting Information Figure S4. Chloroplast inhibitors have limited effect on light-induced cotyledon expansion, but affect plasmodesmata formation. The effect of norflurazon (NF), lincomycin (Linco) and DCMU were tested. (**a**) Scanning electron micrographs of entire cotyledon cross sections of *G. gynandra* seedlings, 0 h and 48 h after light induction. (**b**) Representative scanning electron micrographs of M-BS interfaces of 0 h and 48 h during de-etiolation of *G. gynandra* cotyledons treated with NF, Linco and DCMU, as well as untreated seedlings (Control). Red arrows indicate individual plasmodesma. Scale bar = 1 μm

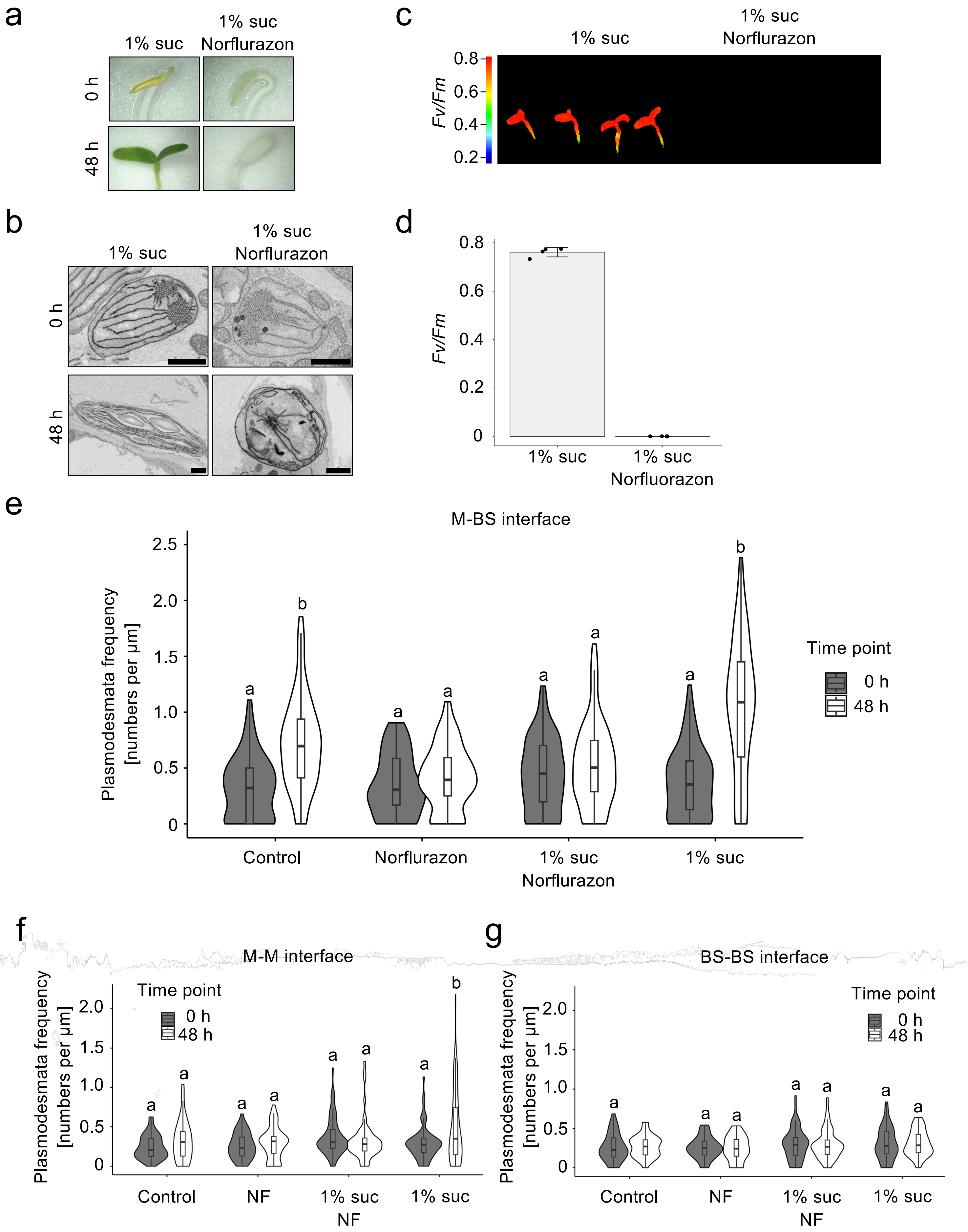

a
1% suc
1% suc Norflurazon
0 h
48 h
b
1% suc
1% suc Norflurazon
0 h
48 h
c
1% suc
1% suc Norflurazon
Fv/Fm
0.8
0.6
0.4
0.2
d
Fv/Fm
0.8
0.6
0.4
0.2
0
1% suc
1% suc Norfluorazon
e
M-BS interface
Plasmodesmata frequency [numbers per µm]
2.5
2.0
1.5
1.0
0.5
0
a
b
a
a
a
a
a
b
Control
Norflurazon
1% suc Norflurazon
1% suc
Time point
0 h
48 h
f
M-M interface
Plasmodesmata frequency [numbers per µm]
Time point
0 h
48 h
2.0
1.5
1.0
0.5
0
Control
NF
1% suc NF
1% suc
g
BS-BS interface
Plasmodesmata frequency [numbers per µm]
Time point
0 h
48 h
2.0
1.5
1.0
0.5
0
Control
NF
1% suc NF
1% suc

Supporting Information Figure S5. Sucrose cannot rescue norflurazon (NF) mediated inhibition of plasmodesmata formation at the Mesophyll (M) and Bundle Sheath (BS) cell interface. (a) Representative images of norflurazon-treated and untreated *G. gynandra* seedlings during de-etiolation (at 0 h and 48 h) with exogenous 1% (w/v) sucrose. (**b**) Scanning electron micrographs of mesophyll etioplasts (0 h) and mature chloroplasts (48 h) of norflurazon-treated and untreated *G. gynandra* seedlings with exogenous 1% (w/v) sucrose. Scale bar represents 1 µm **(c)** Chlorophyll fluorescence images of maximum quantum efficiency of PSII photochemistry (*Fv/Fm*) from 48 h de-etiolated, untreated and norflurazon-treated seedlings grown on 1% (w/v) sucrose. **(d)** *Fv/Fm* measured in *G. gynandra* 48 h after light induction. Bars represent mean ± standard deviation from n = 4 individual seedlings, dots represent individual data points. (**e-g**) Plasmodesmata frequency per µm cell interfaces in *G. gynandra* cotyledons quantified during the dark to light transition (0 h and 48 h time point) and norflurazon treatment, with and without additional 1% (w/v) sucrose supply using high-resolution 2D SEM maps: (**c**) M-M, (**d**) M-BS and (**e**) BS-BS. All interfaces were quantified from cotyledon samples of at least 3 individual seedlings (biological replicates) per time point. Data for control and norflurazon samples grown without exogenous sucrose were replotted from Figure **5e-g.** (**e**) For M-BS interface: 0 h 1% suc NF n = 112, 0 h 1% suc n = 90, 48 h 1% suc NF n = 90, 48 h 1% suc n = 81, cell interfaces were quantified. (**f**) For M-M interface 0 h 1% suc NF n = 73, 0 h 1% suc n = 68, 48 h 1% suc NF n = 62, 48 h 1% suc n = 63 cell interfaces were quantified. (**g**) For BS-BS interface: 0 h 1% suc NF n = 65, 0 h 1% suc n = 53, 48 h 1% suc NF n = 66, 48 h 1% suc n = 44 cell interfaces were quantified. The box and whiskers represent the 25 to 75 percentile and minimum-maximum distributions of the data. Letters show the statistical ranking, pairwise comparison of 0h and 48 h time point for each treatment, using a *post hoc* Tukey test (different letters indicate statistically significant differences at $P < 0.05$). Values indicated by the same letter are not statistically different.

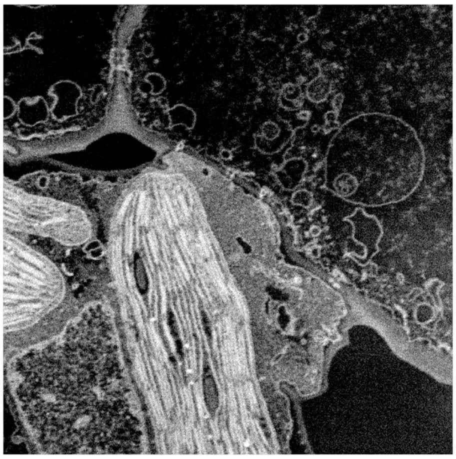

Supporting Information Video S1. 3D reconstruction video of Mesophyll (M) and Bundle Sheath (BS) cell interface in a mature *G. gynandra* leaf. Video was compiled from 80 selected sequential 50 nm sections of M-BS cell interface. In the 3D reconstruction, the cell wall is shown in yellow while plasmodesmata are shown in blue. Video shows BS cell on top and M cells on bottom.

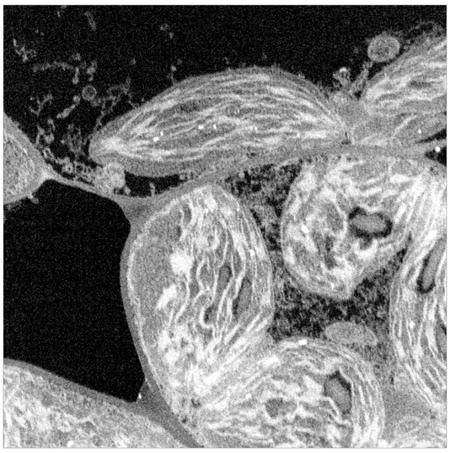

Supporting Information Video S2. 3D reconstruction video of Mesophyll (M) and Bundle Sheath (BS) cell interface in a mature *T. hassleriana* leaf. Video was compiled from 80 selected sequential 50 nm sections of M-BS cell interface. In the 3D reconstruction, the cell wall is shown in yellow while plasmodesmata are shown in blue. Video shows BS cell on top and M cells on bottom.

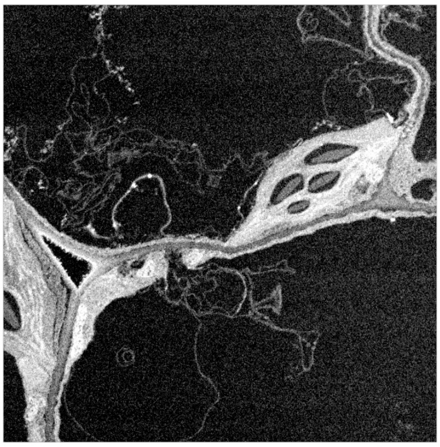

Supporting Information Video S3. 3D reconstruction video of Mesophyll (M) and Bundle Sheath (BS) cell interface in a mature *A. thaliana* leaf. Video was compiled from 80 selected sequential 50 nm sections of M-BS cell interface. In the 3D reconstruction, the cell wall is shown in yellow while plasmodesmata are shown in blue. Video shows BS cell on top and M cells on bottom.

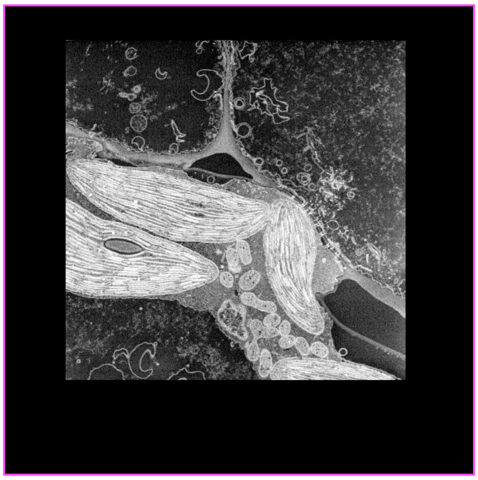

Supporting Information Video S4. Compiled video of sequential 50 nm sections of Mesophyll (M) and Bundle Sheath (BS) cell interface in mature leaves of C_4 *G. gynandra.* *n* = 281 sequential sections; *N* = 467 individual plasmodesmal frequencies measured (some sections contained more than one M-BS interface). Video shows BS cells on top and M cells on bottom.

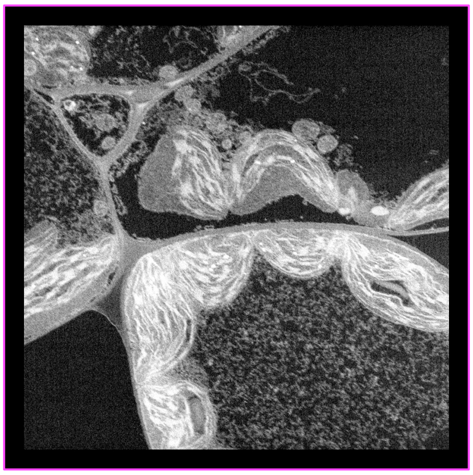

Supporting Information Video S5. Compiled video of sequential 50 nm sections of Mesophyll (M) and Bundle Sheath (BS) cell interface in mature leaves of C_3 *T. hassleriana*. *n* = 367 sequential sections; *N* = 367 individual plasmodesmal frequencies measured. Video shows BS cells on top and M cells on bottom.

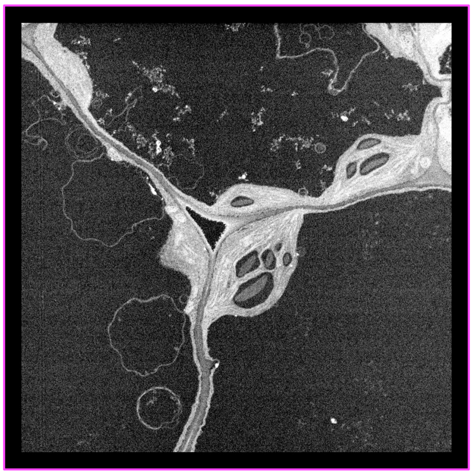

Supporting Information Video S6. Compiled video of sequential 50 nm sections of Mesophyll (M) and Bundle Sheath (BS) cell interface in mature leaves of C_3 *A. thaliana.* *n* = 438 sequential sections; *N* = 886 individual plasmodesmal frequencies measured. Video shows BS cells on top and M cells on bottom.
